# Study on the construction of algal–bacterial consortium comprising *Pantoea vagans* ZHS-1 and *Nostoc* sp. GD2 and its growth-promoting effect on rice

**DOI:** 10.1128/spectrum.03506-25

**Published:** 2026-08-31

**Authors:** Ji Dong, Chengcai Yue, Shiqi Tian, Xueying Zhou, Siyuan Li, Dongchao Wang, Zhihai Wu, Meiying Yang, Xue Yang, Lei Wu

**Affiliations:** 1 College of Life Sciences, Jilin Agricultural University 85112, Changchun, Jilin, China; 2 Faculty of agronomy, Jilin Agricultural University 85112, Changchun, Jilin, China; Connecticut Agricultural Experiment Station, New Haven, Connecticut, USA; Universidade de Brasilia Programa de Pos-Graduacao em Linguistica, Patrolina, Brazil

**Keywords:** algal–bacterial consortium, *Pantoea vagans*, *Desmonostoc muscorum*, plant growth promotion, transcriptome analysis, soil nutrient activation, sustainable agriculture

## Abstract

Excessive use of chemical fertilizers has led to severe agricultural pollution and soil degradation. Algal–bacterial consortia, as eco-friendly microbial inoculants, offer a promising strategy to reduce fertilizer dependency and promote sustainable agriculture. In this study, we constructed a consortium (ZD-1) comprising the potassium-solubilizing bacterium *Pantoea vagans* ZHS-1 and the nitrogen-fixing cyanobacterium *Desmonostoc muscorum* GD2. Co-cultivation at various ratios showed that a 1:1 ratio maximized mutual growth promotion, chemotaxis, and biofilm formation. Under this optimal ratio, the consortium exhibited significantly increased total nitrogen content in BG11 supernatant, as well as enhanced soluble phosphorus and soluble potassium accumulation in specific media containing insoluble P/K sources; these changes were concomitant with upregulation of key functional genes revealed by transcriptomic analysis. Pot experiments demonstrated that ZD-1 inoculation markedly improved rice growth, increased nitrogen, phosphorus, and potassium accumulation in plants, and elevated soil available nutrient contents. Notably, the growth-promoting effect of ZD-1 was comparable to that of full chemical fertilization, and its combination with half-dose fertilizers achieved synergistic yield improvement. Our findings elucidate the molecular interplay between ZHS-1 and GD2 and highlight the potential of the ZD-1 consortium as a viable biofertilizer to partially substitute chemical fertilizers in rice cultivation.

**IMPORTANCE:**

This work establishes an optimal algal–bacterial consortium (ZD-1, 1:1 ratio) composed of *Pantoea vagans* ZHS-1 and *Desmonostoc muscorum* GD2 with clearly defined synergistic mechanisms. By integrating physiological, biochemical, and transcriptomic analyses, we demonstrate that ZD-1 enhances nutrient transformation, biofilm formation, and plant growth. The consortium effectively increases soil available N, P, and K and substitutes a substantial portion of chemical fertilizers while maintaining rice yield. This study provides a robust theoretical and practical basis for developing green microbial fertilizers and advancing sustainable agriculture.

## INTRODUCTION

Global population growth, rising food demand, shrinking arable land, and increasing environmental pressures have posed significant challenges to modern agriculture (1), necessitating the innovation of sustainable agricultural technologies that enhance productivity while reducing ecological footprints (2). China consumes more than 30% of the world’s agricultural chemicals yet possesses only 9% of the global arable land (3). The low use efficiency and high loss rate of these agrochemicals have caused severe environmental pollution (4). Although the government has implemented measures such as the removal of fertilizer subsidies, the outcomes have been limited, and issues such as soil degradation persist (5).

Microbial fertilizers, often referred to as “green fertilizer,” contain beneficial microorganisms, including cyanobacteria and plant-growth-promoting rhizobacteria (6). They can be applied alone or in combination with chemical fertilizers to partially replace them (7). These biofertilizers contribute to soil improvement (increasing organic matter, enhancing aeration and water retention) (8), nutrient transformation (decomposing organic matter for crop absorption, activating medium and trace elements) (9), and microecological restoration (suppressing pathogenic microorganisms, strengthening nutrient cycling) (10). Moreover, they reduce chemical fertilizer input, stabilize crop yields, and support the development of eco-friendly agriculture.

Recent studies have highlighted the potential of algae-based fertilizers in sustainable crop production. Nguyen et al. demonstrated that algal fertilizers provide crops with nitrogen, phosphorus, and potassium through mild and sustained nutrient release: within 4 weeks, 63.5% of nitrogen (as ammonium-N + nitrate-N) and 25% of phosphorus (as phosphate) were released (11). Certain cyanobacteria can also convert atmospheric nitrogen into plant-available forms via biological nitrogen fixation and secrete phytohormones (e.g., auxins and cytokinins) as well as bioactive substances such as polysaccharides and phenolic compounds (11). Yu et al. reported that algal fertilizers act as highly effective slow-release phosphorus sources, increasing soil Olsen-P content to three times that of chemical phosphorus fertilizer within 90 days and significantly promoting the cycling of carbon, nitrogen, phosphorus, and sulfur in soil (12). Collectively, algal fertilizers offer the dual advantages of slow nutrient release and enhanced soil nutrient availability, with growth-promoting effects on crops comparable to those of chemical phosphorus fertilizers, thereby providing important support for sustainable agricultural fertilization (13, 14).

Despite the promising effects of individual microbial strains, their widespread application in agriculture has been constrained by poor ecological stability and limited adaptability to complex field environments (15). In contrast, composite microbial inoculants often exhibit superior performance. For instance, co-inoculation can increase sweet potato yield (16) and enhance plant tolerance to abiotic stresses (17), while the combination of nitrogen-fixing bacteria and phosphate-solubilizing bacteria has also been shown to improve forage growth and soil quality (18). Therefore, composite microbial inoculants possess greater application and commercialization potential than single-strain inoculants in production practice.

Previously, our laboratory isolated and characterized the nitrogen-fixing cyanobacterium *Desmonostoc muscorum* GD2 and the potassium-solubilizing bacterium *Pantoea vagans* ZHS-1 from paddy soils in Jilin Province, China. GD2 exhibits robust nitrogen-fixing performance, with a maximum nitrogenase activity of 75.33 U·mg⁻¹ protein under optimal conditions, and its application significantly increases total nitrogen and alkali-hydrolyzable nitrogen contents in paddy soil. ZHS-1 secretes substantial amounts of organic acids primarily through the EMP-TCA (Embden–Meyerhof–Parnas–tricarboxylic acid) cycle, with supplementary contributions from pantothenate, glyoxylate, and dicarboxylate metabolic pathways; this organic acid secretion underpins its ability to solubilize insoluble potassium and phosphorus, thereby markedly enhancing available potassium content in the rhizosphere soil (19, 20).

Although both strains individually exhibit functional traits relevant to plant nutrition, their single-strain applications in paddy soil have shown limited and inconsistent effects. Inspired by the ubiquitous mutualistic interactions between microalgae and bacteria in natural ecosystems—including nutrient exchange, signal transduction, and metabolic complementarity—we hypothesized that co-cultivating GD2 and ZHS-1 as a synthetic algal–bacterial consortium could amplify their respective beneficial functions through synergistic interactions.

Accordingly, the present study aimed to (i) determine the optimal co-culture ratio for maximal mutual growth promotion and nutrient-mobilizing performance; (ii) elucidate the material basis and molecular mechanisms underlying consortium formation through chemotaxis, biofilm, and transcriptomic analyses; and (iii) evaluate the efficacy of the optimized consortium in promoting rice growth and activating soil nitrogen, phosphorus, and potassium pools. This work provides a theoretical foundation for understanding algal–bacterial interactions and offers a practical strategy for developing effective microbial fertilizers to reduce chemical fertilizer dependency in rice cultivation.

## MATERIALS AND METHODS

### Experimental strains and culture media

The potassium-solubilizing bacterium strain ZHS-1 and nitrogen-fixing cyanobacterium strain GD2 used in this experiment were obtained from the College of Life Sciences, Jilin Agricultural University. Strain ZHS-1 is elliptical rod shaped and identified as *Pantoea vagans*, which possesses multiple functions of potassium solubilization, phosphate solubilization, and indole-3-acetic acid (IAA) secretion. Strain GD2 is a filamentous cyanobacterium identified as *Desmonostoc muscorum*, with a maximum nitrogenase activity of 75.33 U·mg⁻¹ under optimal conditions, exhibiting a significant nitrogen-fixing function. Strain ZHS-1 was inoculated in Luria–Bertani (LB) and subjected to expanded cultivation under standard culture conditions (30°C, 150 rpm). Nitrogen-fixing cyanobacterium GD2 was inoculated into BG11 liquid medium, placed in a light incubator, and underwent expanded cultivation under standard culture conditions (25°C, light intensity of 2,500 lx, light-dark cycle of 16 h:8 h), with flask shaking 4–6 times at a fixed time every day. The liquid inorganic phosphorus medium consisted of 10 g/L glucose, 5 g/L tricalcium phosphate, 5 g/L magnesium chloride hexahydrate, 0.25 g/L magnesium sulfate heptahydrate, 0.2 g/L potassium chloride (KCl), and 0.1 g/L ammonium sulfate. The pH was adjusted to 7.0, and the medium was autoclaved at 121°C for 20 min before use. The modified potassium-solubilizing medium was composed of 5 g/L glucose, 0.5 g/L yeast extract, 2 g/L disodium hydrogen phosphate, 0.5 g/L ammonium sulfate, 0.3 g/L magnesium sulfate heptahydrate, 0.03 g/L manganese sulfate heptahydrate, 0.03 g/L iron sulfate heptahydrate, 2 g/L potassium feldspar powder, and 15 g/L agar. The pH was adjusted to 7.2, and the medium was autoclaved at 121°C for 20 min prior to use.

### Construction of the algal–bacterial consortium

#### Algae–bacteria co-cultivation experiment

The ZHS-1 bacterial suspension was inoculated into 100 mL of LB medium at a 1% inoculation ratio and cultured under standard conditions until the OD₆₀₀ reached 0.8. It was then centrifuged at 12,000 rpm for 5 min, followed by resuspension in 100 mL of BG11 medium to prepare the ZHS-1 bacterial stock solution for subsequent use. The GD2 algal suspension was inoculated into 100 mL of BG11 liquid medium at a 1% inoculation ratio, placed in a light incubator, and cultured under standard conditions with flask shaking every 12 h until the OD₆₈₀ reached 0.8. It was then centrifuged at 12,000 rpm for 5 min, and the algal cells were collected for subsequent use. A 1 mL aliquot of the ZHS-1 bacterial stock solution was centrifuged at 12,000 rpm for 5 min.

To evaluate the synergistic effects of ZHS-1 and GD2, five mass ratios (ZHS-1:GD2 = 1:1, 1:2, 1:3, 1:4, and 1:5) were tested. The ratios were designed with a higher proportion of GD2 relative to ZHS-1, as algal metabolites typically serve as the primary carbon source for heterotrophic bacteria in algal–bacterial systems. An excessively high proportion of bacteria could lead to intensified competition for nutrients—such as nitrogen, phosphorus, and algal-derived organic carbon—potentially causing nutrient deficiency and growth inhibition of algae, and even disrupting the stability of the consortium (21). The mixed cultures were inoculated into 100 mL BG11 liquid medium and incubated under the standard algal culture conditions for 7 days, with manual shaking every 12 h. The growth status of strain ZHS-1 in the algae–bacteria co-culture solution was quantified using the gradient dilution plate counting method. The growth of algae in the co-culture solution was represented by their chlorophyll content, determined through the following steps: the algal suspension was filtered via a microfiltration membrane with a pore size of 0.45 μm, and the membrane was then transferred to a 10 mL centrifuge tube. Seven milliliters of pre-cooled 80% acetone (analytical grade) was added; after thorough shaking, the mixture was allowed to stand at 4°C in the dark for 24 h. It was then centrifuged at 6,000 rpm and 4°C for 10 min, and the absorbance of the supernatant at 663 nm, 645 nm, and 630 nm was measured using a visible spectrophotometer. All the above operations were performed under dark conditions. The chlorophyll content was calculated using the following formula:

Chla = 11.64 × OD_663_ − 2.16 × OD_645_ − 0.1 **×** OD_630_.

#### Screening for the optimal co-culture ratio

The nitrogen, phosphorus, and potassium contents in the supernatant of co-cultures were measured to evaluate nutrient turnover capacity under the given culture conditions. These measurements reflect the net release of nutrients into the medium under different culture conditions. For phosphorus and potassium analyses, the media contained insoluble P/K sources, allowing assessment of solubilization capacity. For nitrogen analysis in BG11 medium, the measured accumulation results from combined processes including metabolite release and cellular turnover.

For nitrogen content analysis, the mixtures were inoculated into BG11 liquid medium and co-cultured for 7 days under the standard conditions for algae. The total nitrogen concentration in the supernatant was measured by the micro-Kjeldahl method, with the calculation formula as follows:

Nitrogen content (mg/L) = (*V*₁ − *V*₂) × 14 × 1,000/*V*.

Where, *V*₁ represents the volume of standard hydrochloric acid (HCl) consumed in sample titration (mL), *V*₂ represents the volume of standard hydrochloric acid consumed in blank control titration (mL), 14 is the mass of nitrogen per millimole (mg/mmol), and *V* represents the volume of the sample taken (mL).

Determination of phosphorus-solubilizing capacity: A series of standard phosphorus gradient solutions was prepared using a 10 mg/L potassium dihydrogen phosphate (KH₂PO₄) solution. An equal volume of phosphorus-determining reagent was added to each standard solution, followed by incubation in a 45°C water bath for 20 min. After cooling, the absorbance at 660 nm was measured, and a standard curve between phosphorus concentration and absorbance was plotted. A 1 mL aliquot of the ZHS-1 bacterial stock solution was centrifuged at 12,000 rpm for 5 min. Strain ZHS-1 and nitrogen-fixing cyanobacterium strain GD2 were then mixed at mass ratios of 1:1, 1:2, 1:3, 1:4, and 1:5, respectively, and inoculated into 100 mL of liquid inorganic phosphorus medium, followed by co-cultivation for 7 days under the standard culture conditions for bacteria. Every 1 day, 2 mL of algae–bacteria co-culture solution with different ratios was sampled separately, centrifuged at 12,000 rpm for 5 min, and 1 mL of the supernatant was collected and treated according to the aforementioned method. Using the uninoculated medium as a control, the absorbance at 660 nm was measured, and the value was substituted into the standard curve to calculate the soluble phosphorus content in the algae–bacteria co-culture solutions with different ratios.

Determination of potassium-solubilizing capacity: A standard potassium solution was prepared using potassium chloride (analytical grade, dried at 110°C for 2 h). The soluble potassium content in the supernatant was determined using an F-100 flame photometer (Shanghai Yuanxi Instrument Co., Ltd.), and a standard curve between potassium concentration and absorbance was plotted. A 1 mL aliquot of the ZHS-1 bacterial stock solution was centrifuged at 12,000 rpm for 5 min. Strain ZHS-1 and nitrogen-fixing cyanobacterium strain GD2 were then mixed at mass ratios of 1:1, 1:2, 1:3, 1:4, and 1:5, respectively, and inoculated into 100 mL of modified potassium-solubilizing medium, followed by co-cultivation for 7 days under the standard culture conditions for algae. Every day, 2 mL of algae–bacteria co-culture solutions with different ratios was sampled separately, centrifuged at 8,000 rpm for 10 min to collect the supernatant, and the pH value and soluble potassium content of the supernatant were determined.

### Material basis for the formation of the algal–bacterial consortium

#### Quantification of extracellular polysaccharide

To evaluate the potential of ZD-1 in biofilm formation and intercellular adhesion, extracellular polysaccharides—a major component of extracellular polymeric substances (EPSs)—were quantified. The phenol-sulfuric acid method (22) was used to determine the content of extracellular polysaccharide. A standard glucose solution was prepared and subjected to gradient dilution. An equal volume of 0.6% phenol solution was added to each diluted solution, and after mixing well, an equal volume of concentrated sulfuric acid (H₂SO₄) was added rapidly. After thorough mixing, the mixture was incubated in a 30°C water bath for 30 min and then rapidly cooled to room temperature. The absorbance of the solution at 490 nm was measured using a spectrophotometer, and a standard curve between glucose concentration and absorbance was plotted. Strain ZHS-1 and GD2 were used as controls, respectively. Based on the above screening conditions, the optimal ratio of ZHS-1 to GD2 (designated as ZD-1) was selected and inoculated into 100 mL of BG11 liquid medium. Every 3 h, 5 mL of the algae–bacteria co-culture solution was sampled, mixed with twice the volume of absolute ethanol, and allowed to stand at 4°C for 12 h. The precipitate was collected by centrifugation at 12,000 rpm for 15 min. After resuspending the precipitate in 1 mL of ddH₂O, the absorbance at 490 nm was measured using the aforementioned phenol-sulfuric acid method, and the extracellular polysaccharide content was calculated based on the standard curve.

#### Quantification of extracellular proteins

To further evaluate the role of extracellular components in biofilm formation and intercellular adhesion, extracellular protein content—another major constituent of EPS—was quantified using the Coomassie Brilliant Blue method. A standard protein solution was prepared with bovine serum albumin, followed by gradient dilution. Coomassie Brilliant Blue reagent R250 was added to each diluted solution; after mixing well, the mixture was allowed to stand at room temperature for 5 min. The absorbance at 595 nm was measured using a spectrophotometer, and a standard curve between protein concentration and absorbance was plotted. Using strain ZHS-1 and GD2 as controls, respectively, ZD-1 was inoculated into 100 mL of BG11 liquid medium. Every 1 day, 5 mL of the algae–bacteria co-culture solution was sampled separately and centrifuged at 12,000 rpm for 15 min to collect the culture supernatant. The culture supernatant was mixed with Coomassie Brilliant Blue reagent and allowed to stand at room temperature for 5 min. The absorbance at 595 nm was measured, and the extracellular protein content was calculated based on the standard curve.

#### Biofilm formation assay

Biofilm formation was assessed using the crystal violet staining method (23). ZHS-1, GD2, and ZD-1 were resuspended in BG11 liquid medium and inoculated into a 96-well microtiter plate, followed by incubation at 25°C for 3 days. Subsequently, the culture supernatant was gently removed, and the wells were rinsed gently with distilled water. A 0.1% (wt/vol) crystal violet solution was added to the wells to stain the biofilms, and excess crystal violet was washed off with distilled water. The crystal violet bound to the biofilms in the wells was dissolved with 200 μL of absolute ethanol, and the absorbance at 590 nm was measured. Since the absorbance value is positively correlated with the amount of biofilm, the absorbance values were used to compare the differences in biofilm formation among the three groups.

#### Chemotaxis assay

A chemotaxis assay was conducted to evaluate the attraction of ZHS-1 to GD2 exudates and various carbohydrate compounds. Strain ZHS-1 was cultured under standard conditions until the optical density at 600 nm (OD₆₀₀) reached 0.8. The bacterial cells were collected by centrifugation at 12,000 rpm for 10 min and then resuspended in an equal volume of chemotaxis buffer (composed of 4.4 g/L K₂HPO₄, 4.2 g/L KH₂PO₄, and 7.45 mg/L EDTA-Na₂). Sterile-filtered GD2 culture filtrate, as well as 1 M solutions of glucose, sucrose, maltose, trehalose, rhamnose, mannose, mannitol, and betaine prepared in BG11 liquid medium, respectively, were used as chemotactic solutions. With BG11 liquid medium as the control, 100 μL of each chemotactic solution was aspirated using a 1 mL sterile syringe. The needle of the syringe was tightly inserted into the tip of a 200 μL pipette that had already aspirated 100 μL of the resuspended bacterial solution, ensuring the interface was completely sealed. The two solutions were brought into full contact and incubated at 25°C for 120 min. Bacterial counting of the solution inside the needle was performed using the gradient dilution plate method. The solution inside the needle was subjected to gradient dilution and inoculated onto LB solid medium, followed by incubation at 30°C for 2–3 days. The growth of colonies was observed and recorded. The relative chemotaxis index (RCI) was used to characterize the chemotactic ability of the strain toward the aforementioned substances. It is defined as the ratio of the average colony-forming units (CFUs) in the treated group to the CFUs in the control group. Generally, if RCI ≥ 2, the chemotaxis of the treated group is considered to have a significant difference compared with the control group.

### Transcriptomic analysis and RT-qPCR validation

ZD-1 cultured for 4 days was collected for transcriptome analysis, and sequencing was performed by Beijing Novogene Technology Co., Ltd. The Bowtie2 software was used for genome alignment, and the DESeq2 R package was applied to analyze differentially expressed genes (DEGs), with a *P*-value < 0.05 considered a significant difference. Additionally, GOseq and KOBAS software were used for Gene Ontology (GO) functional enrichment analysis and Kyoto Encyclopedia of Genes and Genomes (KEGG) pathway enrichment analysis, respectively.

Total RNA was extracted using the Trizol method, and then reverse-transcribed into complementary DNA (cDNA) using the PerfectStart Uni RT&qPCR Kit (Beijing TransGen Biotech Co., Ltd.). This cDNA was used for qPCR validation of differentially expressed genes, with 16S rDNA serving as the reference gene. The reverse transcription system, qPCR primers for differentially expressed genes, and reaction conditions are provided in Tables S1 to S4. Statistical analyses were performed using Microsoft Excel 2013 and IBM SPSS Statistics 26. Duncan’s test and independent samples *t*-test were applied for significance testing, with *P* < 0.05 considered statistically significant.

### Effects of the algal–bacterial consortium on rice growth

#### Experimental design and treatments

The local main rice variety “Changjing 616” was selected as the test plant. Soil was collected from the experimental field of Jilin Agricultural University (125°41′E, 43°80′N), with surface residues removed and a sampling depth of 0–20 cm. Opaque plastic buckets were used as pots, with an upper diameter of 30 cm, a lower diameter of 27 cm, and a height of 30 cm. Five treatments were set up: (i) Nitrogen, Phosphorus and Potassium (NPK) fertilizers; (ii) NPK fertilizers + ZD-1; (iii) ZD-1 alone; (iv) N fertilizer + ZHS-1; and (v) PK fertilizers + GD2. Each treatment had five replicates. NPK fertilizers were applied at rates equivalent to 2.14 g N (urea), 3.84 g P₂O₅ (superphosphate), and 0.76 g K₂O (potassium chloride) per pot.

ZD-1 was prepared by co-culturing ZHS-1 and GD2 at a 1:1 ratio for 4 days, centrifuged at 12,000 rpm for 5 min, and resuspended in sterile water. The microbial suspension was applied at 2 mL per planting hole. Each pot contained three holes, with eight seeds sown per hole. Treatments 4 (*N* + ZHS-1) and 5 (PK + GD2) were designed as exploratory groups to provide preliminary insights into the potential role of single strains in partial fertilizer substitution. However, it is important to note that, due to the absence of single-strain controls without fertilizer application, these two treatments cannot be directly and rigorously compared with treatments 1, 2, and 3 to assess the standalone efficacy of the consortium vs individual strains. Therefore, the statistical comparisons and main conclusions regarding the growth-promoting effect of ZD-1 are primarily drawn from treatments 1, 2, and 3. Plants were grown under greenhouse conditions with natural light and irrigated every other day. Samples of rice roots, stems, leaves, and rhizosphere soil were collected at the seedling, filling, and maturity stages for further analysis.

#### Determination of nitrogen, phosphorus, and potassium in rice tissues

At the seedling stage, filling stage, and maturity stage of rice, root, stem, and leaf organs were sampled from the plants, respectively. The root, stem, and leaf samples at each growth stage were subjected to digestion treatment separately. Rice plant samples were placed in an oven at 105°C for drying and deactivation. A 0.5 g portion of the dried rice sample was weighed and added to a digestion tube, followed by the addition of 2 mL distilled water and 8 mL concentrated H₂SO₄. The mixture was allowed to stand overnight. The digestion tube containing the sample was placed in a graphite digester, preheated at 250°C for 10 min, then heated to 400°C and maintained for 40 min. After cooling for 15 min, 5 mL of hydrogen peroxide (H₂O₂) was added. The sample was digested at 400°C for 20 min; after cooling, another 3 mL of H₂O₂ was added, and digestion was continued until the solution became clear. The digested solution was poured into a 100 mL volumetric flask, and distilled water was added to bring it to the marked volume. This solution was used as the test solution.

The distillation method was used to determine the nitrogen content in plants (24): 10 mL of the test solution was taken and distilled using a K9860 automatic Kjeldahl nitrogen analyzer (Haier Future Technology Group Co., Ltd.). After absorption with boric acid, the solution was titrated with 0.01 N sulfuric acid until it turned magenta. The nitrogen content was calculated using the following formula based on the consumption of sulfuric acid:

Nitrogen content = *N* × (*V* – *V*₀) × 0.014/*W* × 100.

Where, *N* represents the standard normality of sulfuric acid, *V* denotes the standard volume of sulfuric acid consumed in the determination, *V*₀ stands for the volume of sulfuric acid consumed in the blank determination, and *W* is the weight of the sample.

The vanadium-molybdenum yellow colorimetric method was used to determine the phosphorus content in plants (25). A phosphorus standard solution was prepared using KH₂PO₄. After gradient dilution and neutralization, 10 mL of ammonium vanadomolybdate reagent was added, and the volume was made up to 50 mL. After 15 min, colorimetry was performed at a wavelength of 490 nm to draw a standard curve. A 5 mL aliquot of the test solution was taken and developed color following the above steps. The phosphorus concentration was obtained by referring to the standard curve, and the phosphorus content was calculated using the following formula:

Phosphorus content = ρ × *V*₃ × (*V*₁/*V*₂) × 10⁻⁴/*m*.

Where ρ represents the phosphorus concentration obtained from the standard curve, *V*₃ denotes the volume of the colored solution, *V*₁ stands for the constant volume of the digested solution, *V*₂ is the volume of the aliquot taken for determination, and *m* is the mass of the sample. The flame photometric method was used to determine the potassium content in plants. A potassium standard solution was prepared using KCl. Both the standard solution and the test solution were sequentially introduced into an F-100 flame photometer (Shanghai Yuanxi Instrument Co., Ltd.) for determination. The potassium content was calculated based on the standard curve using the following formula:

Potassium content = ρ × *V* × *D*/*W* × 1,000.

Wherein, ρ represents the potassium concentration obtained from the standard curve, *V* is the constant volume of the digestion solution, *D* denotes the aliquot multiple, and *W* stands for the dry weight of the sample.

#### Determination of nitrogen, phosphorus, and potassium in soil

Rice rhizosphere soil was collected, air-dried, and then passed through a 100-mesh sieve to serve as the reserve soil sample. Its nitrogen content (including available nitrogen and total nitrogen), phosphorus content (including available phosphorus and total phosphorus), and potassium content (including available potassium and total potassium) were determined separately. Determination of soil available nitrogen content (26): The diffusion dish method was used. In the outer chamber of the diffusion dish, 2 g of the soil sample and 1 g of ferrous sulfate powder were added. In the inner chamber of the diffusion dish, 2 mL of 2% boric acid solution was quickly added, followed by the dropwise addition of methyl red-bromocresol green mixed indicator. Then, 10 mL of 1.8 mol/L sodium hydroxide (NaOH) solution was added to the outer chamber. After smearing gum arabic on the edge of the dish, it was sealed with ground glass and reacted at a constant temperature of 40°C for 24 h. The inner chamber was titrated with 0.01 mol/L HCl until the solution changed from blue to light red, and the volume of consumed hydrochloric acid was recorded. The available nitrogen content was calculated using the following formula:

Nitrogen content = (*V*_1_ − *V*_0_) × *C* × 14 × 1,000/*m*

Where *V*₁ represents the volume of hydrochloric acid consumed in the sample titration, *V*₀ denotes the volume of hydrochloric acid consumed in the blank titration, *C* is the concentration of the standard hydrochloric acid solution, 14 is the molar mass of nitrogen, 1,000 is the unit conversion factor (converting grams to milligrams), and *m* is the mass of the air-dried soil sample.

Determination of total nitrogen content in soil (27): The Kjeldahl digestion-Kjeldahl nitrogen determination method was adopted. A 1 g portion of the reserve soil sample was placed into a Kjeldahl flask, followed by the addition of 1 mL deionized water, 2 g accelerator (potassium sulfate: copper sulfate: selenium powder = 100:10:1), and 5 mL concentrated H₂SO₄. The sample was digested at 150°C for 10–15 min, then the temperature was increased to 480°C and maintained for 90 min until all black particles turned white or grayish green. After cooling, the digested solution was transferred to a 100 mL volumetric flask and brought to volume. Total nitrogen content was determined using a Kjeldahl nitrogen analyzer (for the control group, an equal amount of silica was used to replace the soil sample).

Determination of soil available phosphorus content: The NaHCO₃ extraction-molybdenum-antimony anti-colorimetric method was used. A 2.5 g portion of the reserve soil sample was placed into a 250 mL phosphorus-free Erlenmeyer flask, followed by the addition of 0.2 g phosphorus-free activated carbon and 50 mL of 0.5 mol/L NaHCO₃ solution. After sealing with parafilm, the mixture was oscillated at a constant temperature for 30 min, then filtered through phosphorus-free filter paper. A 10 mL aliquot of the filtrate was taken, and 35 mL of distilled water and 5 mL of molybdenum-antimony anti-mixed color developer were added. After standing for 30 min for color development, the absorbance was measured at 700 nm.

Determination of total phosphorus content in soil: The solid sodium hydroxide fusion method was employed. A 0.2 g portion of the soil sample was placed into a nickel crucible, moistened with 2–3 drops of absolute ethanol, and then 2.0 g of NaOH was added. The crucible was covered with a lid (leaving a small gap) and placed into a muffle furnace. It was maintained at 200°C for 20 min, then the temperature was increased to 720°C for 25 min of ignition. After cooling, distilled water was added to dissolve the fusion product, and the inner wall of the crucible was rinsed with 3 mol/L H₂SO₄. The solution was transferred to a 100 mL volumetric flask and brought to volume. Total phosphorus content was determined using the molybdenum-antimony anti-colorimetric method (for the blank control, no soil sample was added).

Determination of soil available potassium content: The flame atomic absorption spectrophotometry method was used. A 5.0 g portion of the reserve soil sample was placed into a 250 mL Erlenmeyer flask, and 50 mL of 1 mol/L ammonium acetate solution was added. After oscillating for 30 min, the mixture was filtered through phosphorus-free filter paper, and the filtrate was determined using a flame spectrophotometer.

Determination of total potassium content in soil: The soil samples were treated following the digestion method used for total phosphorus, and their total potassium content was determined by flame photometry.

### Statistical analysis

All data were expressed as mean ± standard deviation (SD) from at least three independent replicates. Statistical analyses were performed using IBM SPSS Statistics 26. Differences among treatments were assessed by one-way analysis of variance followed by Duncan’s multiple range test. A *P*-value < 0.05 was considered statistically significant. Graphs were generated using GraphPad Prism 9.0 and OriginPro 2021.

## RESULTS

### Optimization of the algal–bacterial consortiun

#### Growth dynamics of ZHS-1 and GD2 under co-culture

**Fig 1:**
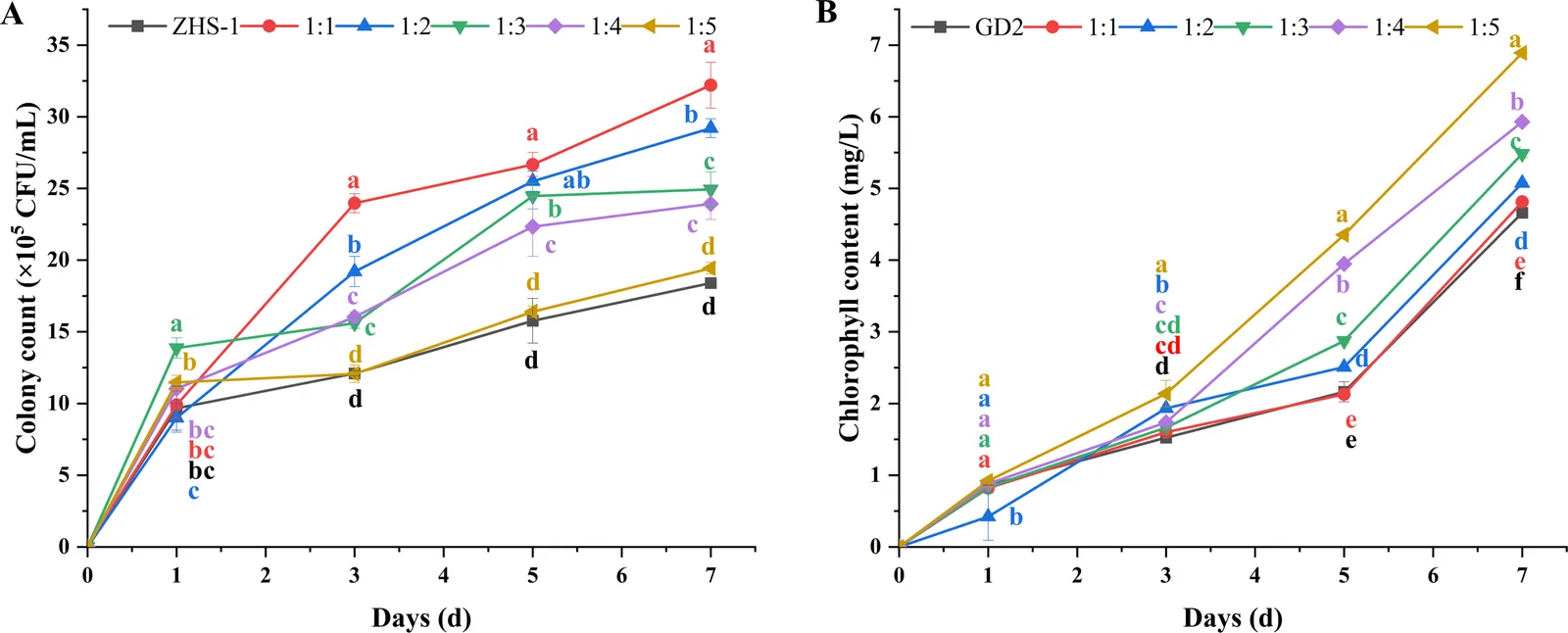
The growth of ZHS-1 and GD2 under different co-culture ratios. (**A**) Colony-forming units (CFU) of ZHS-1. (**B**) Chlorophyll a content of GD2. Data are presented as mean ± SD (*n* = 3). Different letters indicate significant differences (*P* < 0.05).

Co-cultivation of ZHS-1 and GD2 at different ratios revealed no antagonistic effects between the two strains. When the proportion of GD2 was excessively high (bacteria: cyanobacteria = 1:5), the cyanobacteria exerted no significant promoting or inhibitory effect on the bacteria, and the growth of ZHS-1 was comparable to that in pure culture. In contrast, reducing the GD2 proportion significantly enhanced ZHS-1 proliferation, with the 1:1 ratio showing the most pronounced effect: on day 3, the colony count of ZHS-1 reached 26.67 × 10⁵ CFU/mL, which was significantly higher than that of other ratios (Fig. 1A). Under the 1:1 ratio, the chlorophyll content of GD2 was comparable to that of the pure culture, indicating that ZHS-1 did not inhibit algal growth (Fig. 1B). In other ratios, the total chlorophyll content increased with increasing GD2 proportion, reflecting higher initial algal biomass. Based on these results, the 1:1 mass ratio was selected as the optimal condition for subsequent experiments and designated as ZD-1.

#### Nutrient turnover capacity of the consortium

**Fig 2:**
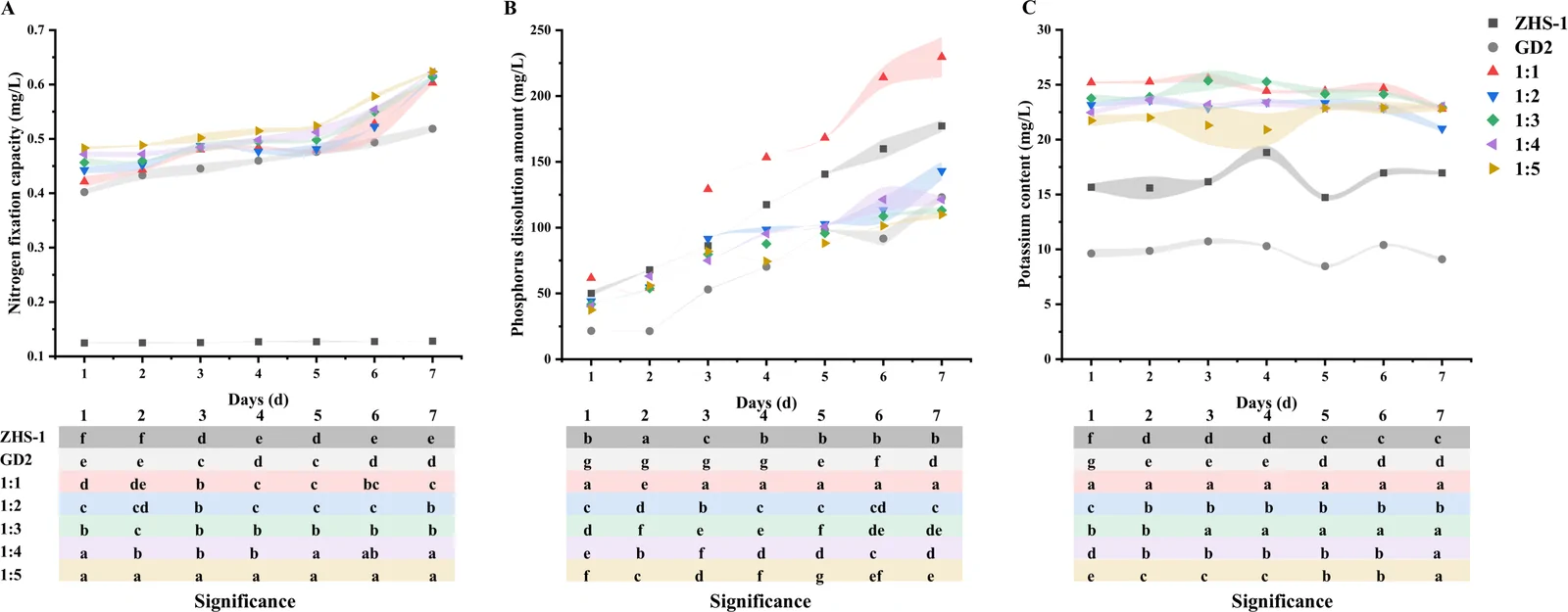
Nutrient turnover capacity of ZHS-1 and GD2 under different co-culture ratios. (**A**) Nitrogen content in supernatant. (**B**) Soluble phosphorus content. (**C**) Soluble potassium content. Data are presented as mean ± SD (*n* = 3). Different letters indicate significant differences (*P* < 0.05).

To evaluate nutrient transformation-related parameters in co-cultures with different ratios, the contents of total nitrogen (in BG11 medium), soluble phosphorus (in IPM), and soluble potassium (in MKSM) were monitored over 7 days (Fig. 2).

As shown in Fig. 2A, total nitrogen concentrations in the supernatants of all co-culture groups increased with cultivation time and were significantly higher than those of the axenic ZHS-1 or GD2 controls. The 1:5 ratio exhibited the highest nitrogen content at day 7, but the increase was not proportional to the biomass input. From the perspective of cost-effectiveness and balanced growth, the 1:1 ratio was considered optimal. Because BG11 medium contains a high concentration of NaNO₃, the observed nitrogen accumulation cannot be directly attributed to biological nitrogen fixation; it likely results from combined processes including metabolite release and cellular turnover.

Soluble phosphorus concentrations measured in IPM (insoluble P source) are shown in Fig. 2B. ZHS-1 monoculture showed strong P-solubilizing activity, whereas GD2 monoculture exhibited negligible activity. Among the co-culture ratios, only the 1:1 consortium displayed significantly higher soluble P than the ZHS-1 control from day 3 onward. Other ratios showed lower soluble P levels, probably because part of the solubilized phosphorus was assimilated by GD2.

Soluble potassium concentrations measured in MKSM (insoluble K source) are presented in Fig. 2C. All co-culture ratios exhibited significantly higher soluble K than the two monoculture controls. The 1:1 ratio showed the highest K-solubilizing activity during the first 6 days; this advantage diminished by day 7, possibly due to increased potassium demand of the rapidly growing ZHS-1 population.

Based on the comprehensive evaluation of mutual growth promotion and nutrient-mobilizing performance, the 1:1 ratio was selected as the optimal consortium and designated ZD-1 for all subsequent experiments.

### Physiological basis of consortium formation

#### Chemotactic response of ZHS-1 to GD2 exudates

**Fig 3:**
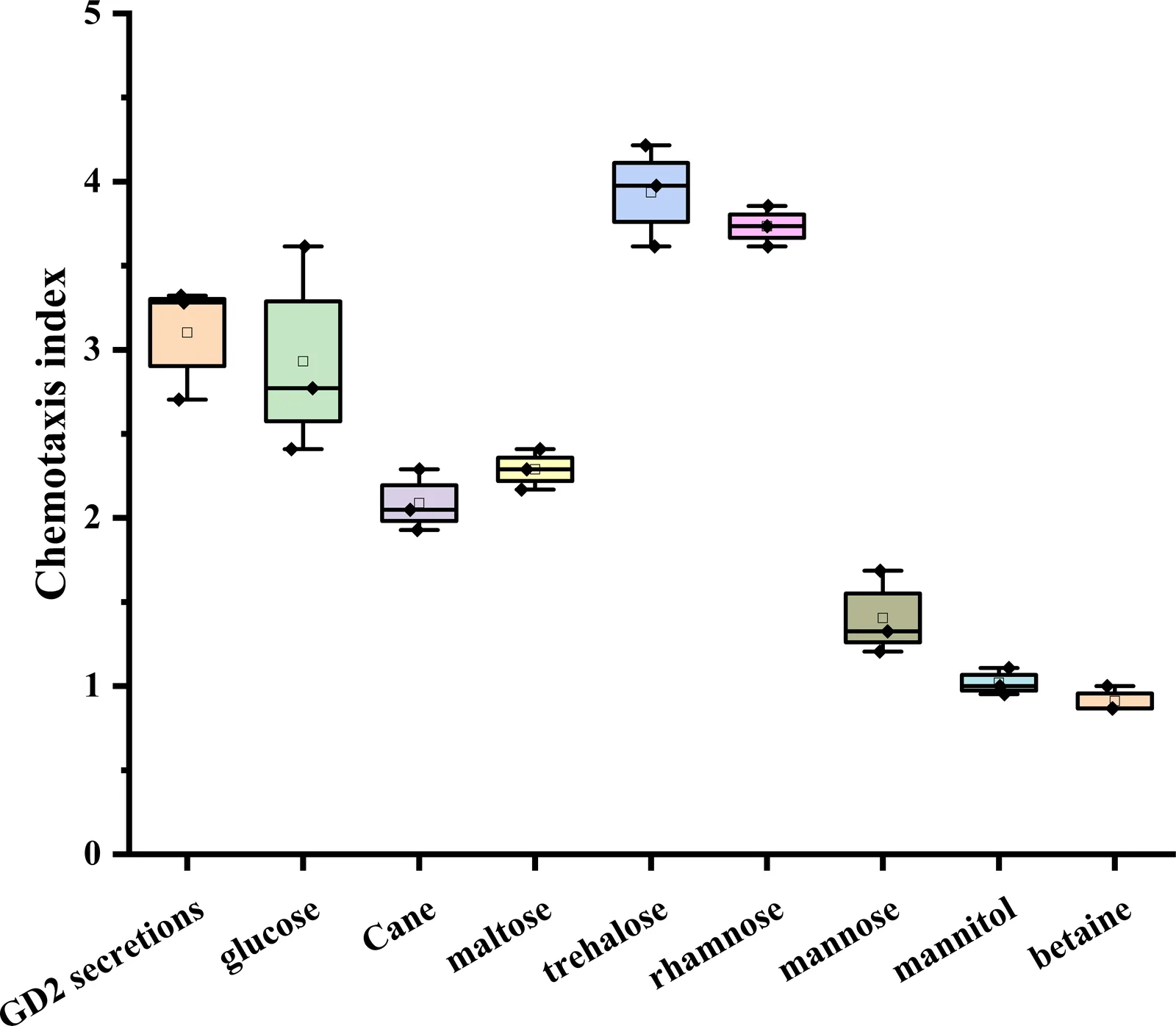
Chemotactic response of ZHS-1 to GD2 exudates and carbohydrate compounds. The relative chemotaxis index (RCI) was calculated as CFU ratio relative to BG11 control. Data are presented as mean ± SD (*n* = 3).

During their growth and metabolism, algae secrete a variety of substances. Since carbohydrates are key limiting factors for bacterial proliferation, it is hypothesized that the secretions of GD2 may contain carbohydrate substances that promote the growth of ZHS-1, which is one of the foundations for the formation of their consortium. To test this hypothesis and to identify potential chemoattractants, the chemotactic response of ZHS-1 to GD2 exudates and eight representative carbohydrate compounds was examined (Fig. 3). Among the eight tested carbohydrate compounds, ZHS-1 showed significant chemotaxis toward trehalose (RCI = 5.2), rhamnose (RCI = 4.1), glucose (RCI = 3.6), sucrose (RCI = 2.8), maltose (RCI = 2.5), and mannose (RCI = 2.3). No significant chemotaxis was observed toward mannitol or betaine (RCI < 2). Notably, trehalose—a disaccharide that is typically metabolized more slowly than monosaccharides such as glucose—exhibited the highest RCI value, while glucose showed a substantially lower RCI. This pattern, where the expected metabolic rate of a compound does not correlate with its ability to attract ZHS-1, strongly supports the interpretation that the observed bacterial accumulation reflects genuine chemotactic migration rather than differential bacterial growth within the test solutions.

ZHS-1 exhibited strong chemotactic responses to GD2 exudates, with an RCI of 4.7 (Fig. 3), which fell between the values for trehalose and rhamnose. This intermediate RCI value is consistent with GD2 exudates being a complex mixture containing multiple chemoattractant compounds, including those identified above. The strong chemotactic index of ZHS-1 toward GD2 secretions confirms the material basis for the formation of the consortium between the two strains and suggests that chemoattraction plays a key role in initiating their symbiotic interaction.

#### Extracellular polysaccharide production, extracellular protein production, and biofilm formation

Biofilms are complex structures composed of EPS such as extracellular DNA, proteins, and polysaccharides (28). They can provide a stable living environment for the algal−bacterial consortium and protect the algal and bacterial cells from interference by physical, chemical, and biological factors in the external environment. Additionally, the porous structure of biofilms can promote material exchange and information transmission between algae and bacteria, which is conducive to their interaction and synergistic growth (29). Therefore, we detected the extracellular polysaccharide content, extracellular protein content, and biofilm formation of the algal−bacterial consortium.

**Fig 4:**
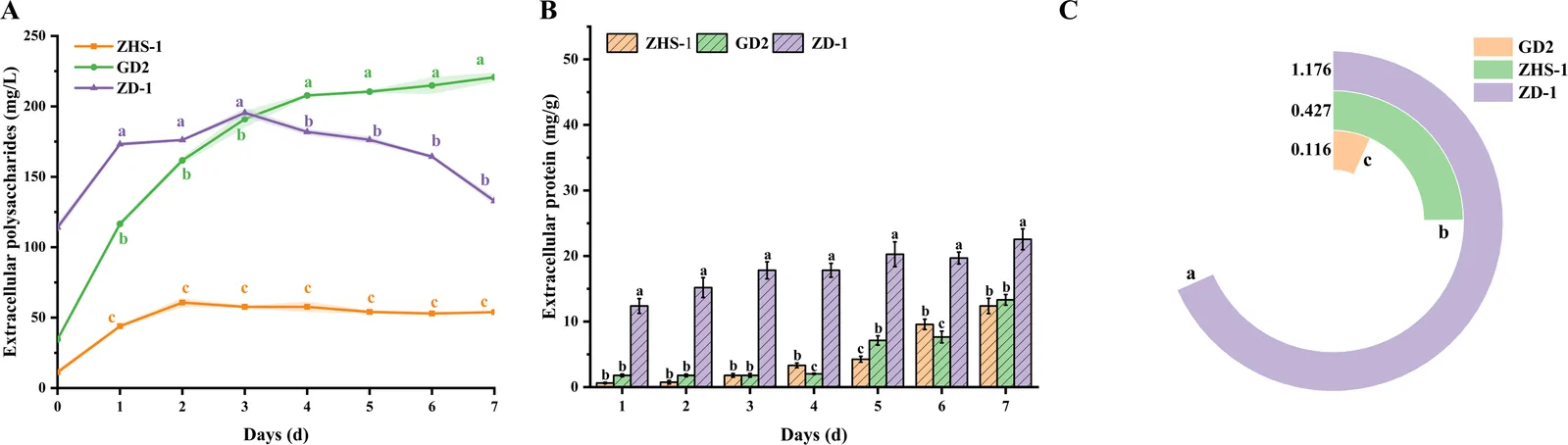
Extracellular polysaccharide production, extracellular protein production, and biofilm formation of ZHS-1, GD2, and ZD-1. (**A**) Extracellular polysaccharide content. (**B**) Extracellular protein content. (**C**) Biofilm formation (OD₅₉₀). Different letters indicate significant differences (*P* < 0.05).

The extracellular polysaccharide content in ZD-1 increased rapidly during the first 72 h, reaching 173.16 mg/L at 24 h—approximately equal to the sum of extracellular polysaccharides produced by ZHS-1 (43.93 mg/L) and GD2 (116.60 mg/L) alone (Fig. 4A). The extracellular polysaccharides peaked at 72 h and declined thereafter, suggesting utilization of algal-derived polysaccharides by ZHS-1 as a carbon source.

The biofilm formation of ZD-1 was significantly superior to that of single strains, with OD₅₉₀ values 4.11- and 15.14-fold higher than those of ZHS-1 and GD2, respectively (Fig. 4C). ZHS-1 exhibited weak biofilm formation due to low extracellular polysaccharide secretion and absence of algal-derived carbon support. GD2, despite high extracellular polysaccharide production, showed limited colonization capacity without bacterial adhesion. These results indicate that ZHS-1 and GD2 synergistically enhance biofilm formation through complementary extracellular polysaccharide production and structural integration.

Similarly, the extracellular protein content of ZD-1 was significantly higher than that of ZHS-1 and GD2 monocultures (Fig. 4B), further confirming enhanced metabolic activity under co-culture conditions.

### Transcriptomic analysis of synergistic interactions

#### Differential gene expression in ZHS-1 under co-culture

**Fig 5:**
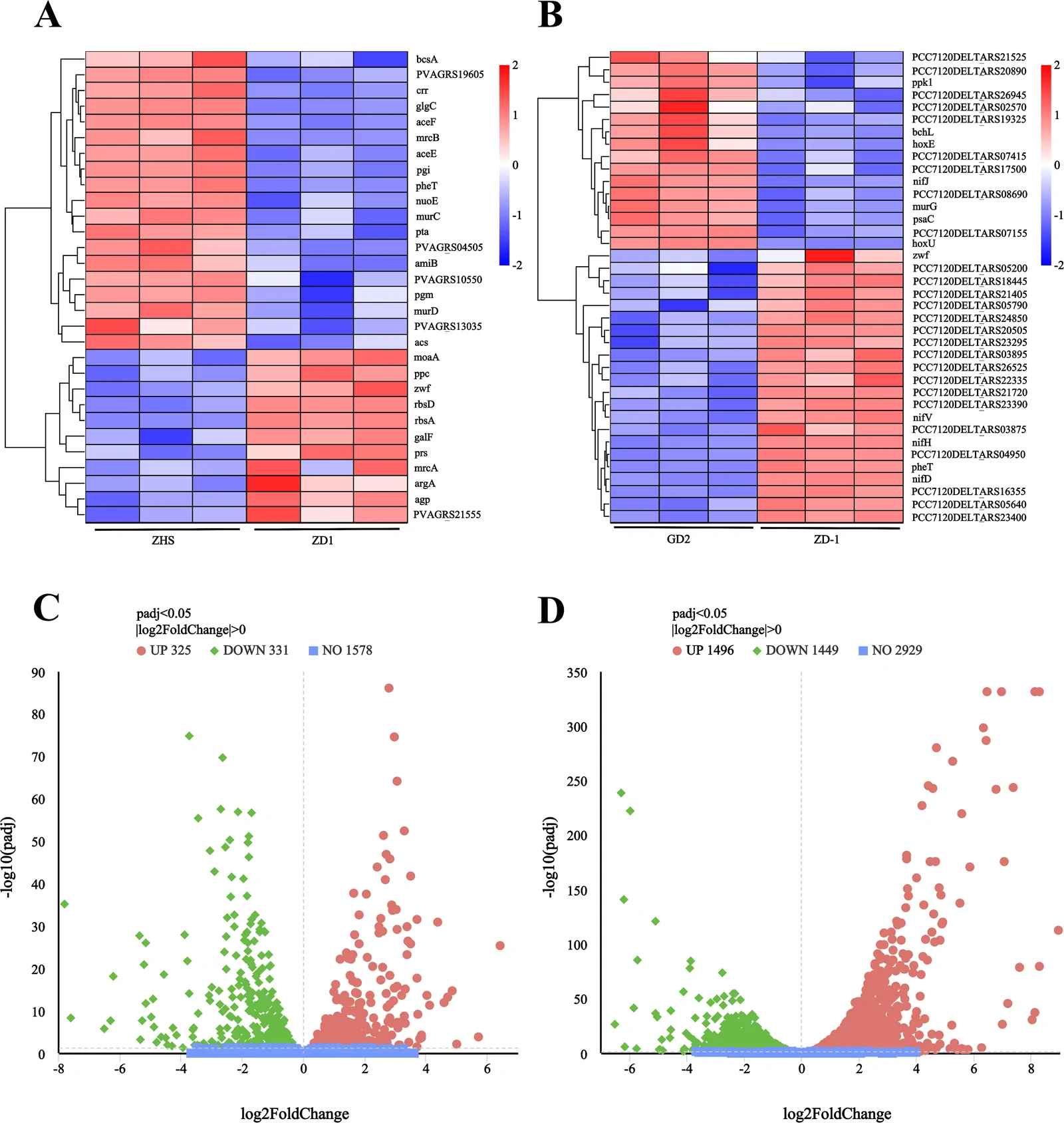
Transcriptomic profiling of ZD-1. (**A**) Volcano plot of DEGs in ZHS-1 (ZD-1 vs ZHS-1 pure culture). (**B**) Volcano plot of DEGs in GD2 (ZD-1 vs GD2 pure culture). (**C**) Heatmap of key DEGs in ZHS-1. (**D**) Heatmap of key DEGs in GD2.

Compared to ZHS-1 pure culture, ZD-1 exhibited 2,234 DEGs in ZHS-1, of which 325 were significantly upregulated and 331 significantly downregulated (|log₂FC| > 1, *P*adj < 0.05; Fig. 5A).

**Fig 6:**
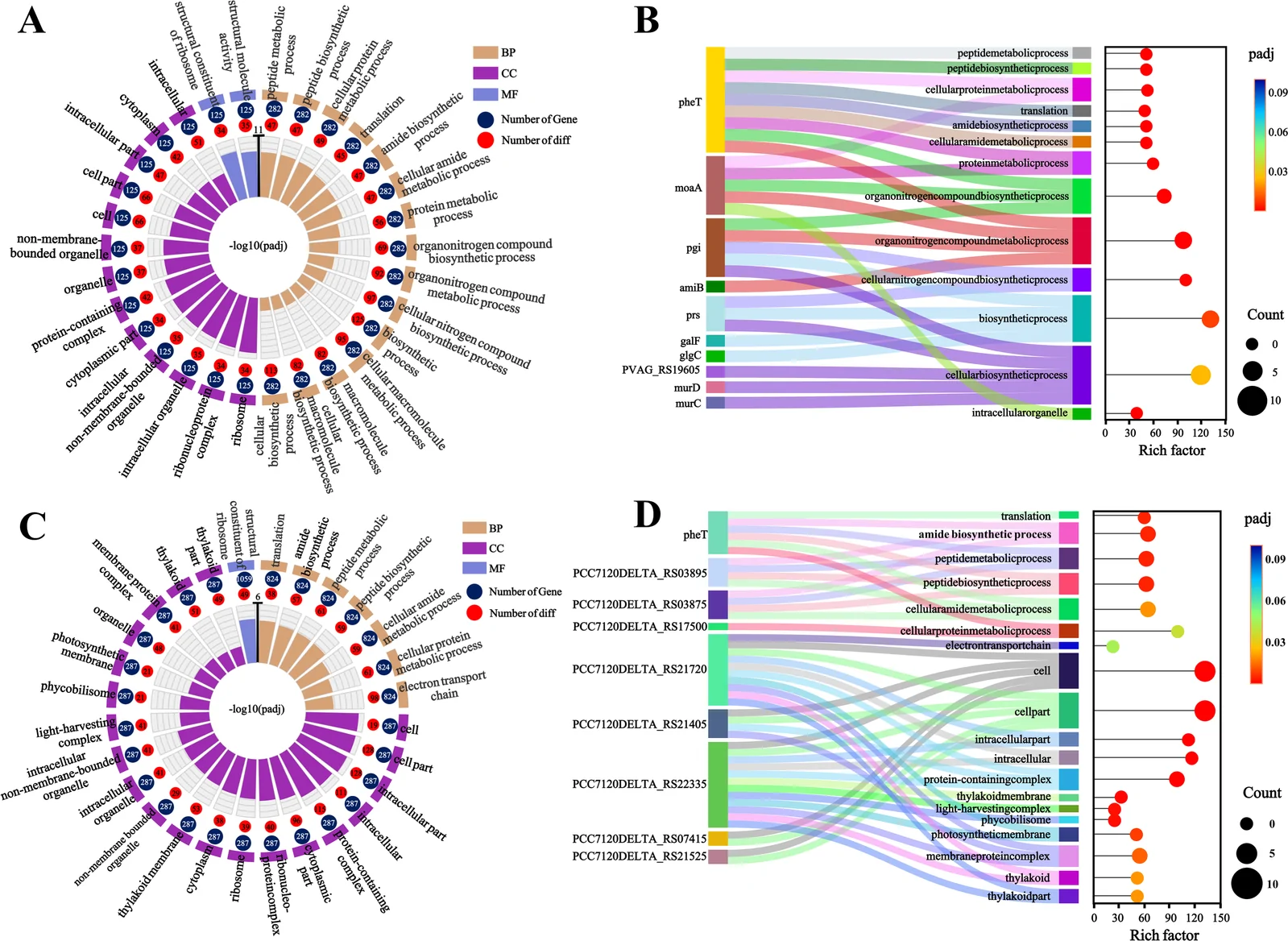
GO enrichment analysis of DEGs. (**A**) GO bar plot of all DEGs in ZHS-1. (**B**) Sankey bubble plot of key DEGs in ZHS-1. (**C**) GO bar plot of all DEGs in GD2. (**D**) Sankey bubble plot of key DEGs in GD2.

GO enrichment analysis showed that upregulated DEGs were significantly enriched in biological processes related to ribosome biogenesis, translation, carbohydrate metabolism, and nucleotide biosynthesis (Fig. 6A and B). Key upregulated genes included: *zwf* gene (glucose-6-phosphate dehydrogenase), *prs* gene (phosphoribosyl pyrophosphate synthase), *galF* gene (UTP-glucose-1-phosphate uridylyltransferase subunit), *ppc* gene (phosphoenolpyruvate carboxylase), and *moaA* gene (molybdenum cofactor biosynthesis protein). The upregulation of the *zwf* gene enables the production of more NADPH and ribose-5-phosphate (30). The upregulation of the *prs* gene promotes the catalysis of ribose-5-phosphate phosphorylation to generate 5-phosphoribosyl-1-pyrophosphate, which is a critical step in purine metabolism and nucleotide biosynthesis (31). The product of the *galF* gene is a component of UTP-glucose-1-phosphate uridylyltransferase, which plays a critical role in polysaccharide synthesis (32). The upregulation of the *galF* gene leads to increased polysaccharide synthesis, which explains why the extracellular polysaccharide content of the bacteria-cyanobacteria consortium is higher than that of single strains. The upregulation of the *ppc* gene enhances the expression of phosphoenolpyruvate carboxylase. When nitrogen sources in the environment are relatively sufficient, the upregulation of the *ppc* gene can optimize carbon skeleton allocation, prioritizing the synthesis of nitrogen-containing compounds (33, 34). As a key gene encoding enzymes for molybdenum cofactor biosynthesis, the upregulation of the *moaA* gene indicates increased synthesis of molybdenum cofactors. This may promote the activity of enzymes dependent on this cofactor, thereby enhancing the consortium’s ability to utilize nitrogen sources in the environment. Meanwhile, the upregulation of the *rbsD* and *rbsA* genes—related to ribose transport—facilitates nucleic acid synthesis and energy metabolism (35). The upregulation of the *mltD* gene enhances the cell’s ability to divide and remodel.

Downregulated DEGs included *glgC* gene (glycogen synthase), *pgi* gene (phosphoglucose isomerase gene), and *amiB* gene (N-acetylmuramyl-L-alanine amidase), indicating reduced glycogen storage and cell wall turnover, consistent with a shift toward active growth and biosynthesis. The *pgi* gene catalyzes the interconversion between glucose-6-phosphate and fructose-6-phosphate, serving as a critical node connecting glycolysis and the pentose phosphate pathway. The downregulation of this gene indicates the enhancement of the pentose phosphate pathway in the bacteria-cyanobacteria consortium, which promotes glucose-6-phosphate to preferentially enter this pathway.

**Fig 7:**
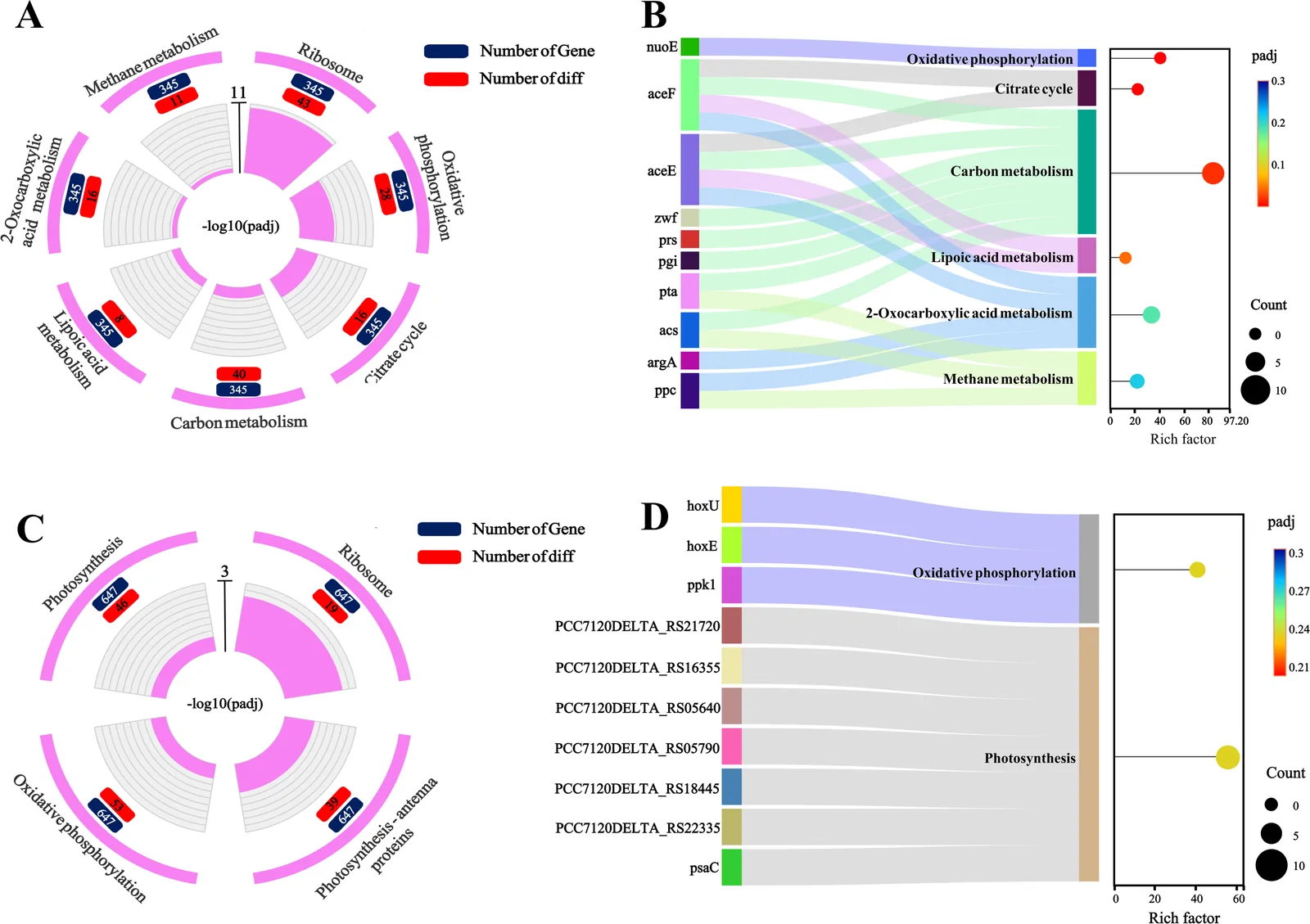
KEGG pathway enrichment analysis of DEGs. (**A**) KEGG bar plot of all DEGs in ZHS-1. (**B**) Sankey bubble plot of key DEGs in ZHS-1. (**C**) KEGG bar plot of all DEGs in GD2. (**D**) Sankey bubble plot of key DEGs in GD2.

KEGG pathway enrichment analysis revealed significant upregulation of ribosome, carbon metabolism, and 2-oxocarboxylic acid metabolism pathways, along with downregulation of the TCA cycle and lipoic acid metabolism (Fig. 7A and B). These changes suggest that ZHS-1, when co-cultured with GD2, shifts from energy storage and oxidative metabolism to rapid growth, nucleotide synthesis, and organic acid production.

#### Differential gene expression in GD2 under co-culture

Compared to GD2 pure culture, ZD-1 exhibited 2,234 DEGs in GD2, with 1,496 significantly upregulated and 1,449 significantly downregulated (Fig. 5B).

GO enrichment analysis showed significant upregulation of photosynthesis, light harvesting, and nitrogen compound biosynthesis pathways (Fig. 6C and D). Key upregulated genes included *nifH* gene (nitrogenase iron protein), *nifD* gene (nitrogenase molybdenum-iron protein alpha subunit), *nifV* gene (homocitrate synthase for FeMo-cofactor biosynthesis), *zwf* gene (glucose-6-phosphate dehydrogenase), and *pheT* gene (phenylalanyl-tRNA synthetase beta subunit). *nifH* encodes nitrogenase iron protein, and *nifD* encodes the α-subunit of nitrogenase molybdenum-iron protein. These two proteins are core components of the nitrogenase complex and directly participate in the key reaction of catalyzing the reduction of nitrogen gas (N₂) to ammonia (NH₃). The significant upregulation of these two genes is a clear marker of nitrogen fixation activation. The *nifV* gene encodes homocitrate synthase, which is involved in the biosynthesis of the iron-molybdenum cofactor of nitrogenase (36). The upregulation of the nifV gene indicates that cyanobacteria (GD2) are not only increasing the synthesis of nitrogenase structural proteins but also enhancing cofactor synthesis to ensure the functionality of nitrogenase, which further supports the efficient operation of nitrogen fixation. This explains why the nitrogen fixation capacity of the bacteria-cyanobacteria consortium is significantly higher than that of single strains. The upregulation of these genes, as determined by RT-qPCR with normalization to reference genes, indicates a true increase in transcriptional activity per cell in the co-culture context, rather than being solely a consequence of increased biomass. The upregulation of the *zwf* gene promotes the encoding of glucose-6-phosphate dehydrogenase, which enhances the activity of the pentose phosphate pathway. This further leads to the massive production of NADPH to meet the high-energy demand of nitrogen fixation. The significant differential expression of these genes indicates that during the interaction between ZHS-1 and GD2, the ability to synthesize carbohydrates and absorb/utilize nitrogenous substances is enhanced, and the growth rate is increased. This further promotes the efficient fixation of nitrogen and the capacity for protein synthesis. The upregulation of the *pheT* gene promotes the encoding of the β-subunit of phenylalanyl-tRNA synthetase—an essential enzyme for the accurate incorporation of amino acids into peptide chains during protein synthesis. This indicates enhanced protein synthesis capacity in cyanobacteria (GD2), which may be associated with the high demand for synthesizing nitrogen fixation-related proteins, supporting rapid protein turnover under nitrogen-fixing conditions. This explains why the contents of extracellular polysaccharides, intracellular proteins, and extracellular proteins in the bacteria-cyanobacteria consortium are higher than those in single *strains*.

Downregulated genes included the *ppk1* gene (polyphosphate kinase), indicating reduced energy storage and a shift toward immediate metabolic utilization of carbon sources. The downregulation of the *ppk1* gene indicates a reduction in the encoding of polyphosphate kinase, which leads to decreased polyphosphate synthesis. This suggests a lower cellular demand for energy storage, potentially due to the abundant carbon sources in the environment—derived from the large quantities of substances such as polysaccharides produced by ZD-1.

KEGG pathway enrichment analysis revealed significant upregulation of photosynthesis and porphyrin/chlorophyll metabolism, along with downregulation of oxidative phosphorylation (Fig. 7C and D). This metabolic shift indicates that GD2, when co-cultured with ZHS-1, enhances photosynthetic energy capture and nitrogen fixation while reducing inefficient respiratory metabolism.

#### RT-qPCR validation

RT-qPCR analysis confirmed the expression patterns of selected DEGs, including *zwf*, *prs*, *galF*, *nifH*, *nifD*, and *nifV*, showing high concordance with RNA-seq data (Fig. S1). These results validate the reliability of the transcriptomic analysis.

### Effects of ZD-1 on rice growth and nutrient uptake

#### Plant growth phenotype

**Fig 8:**
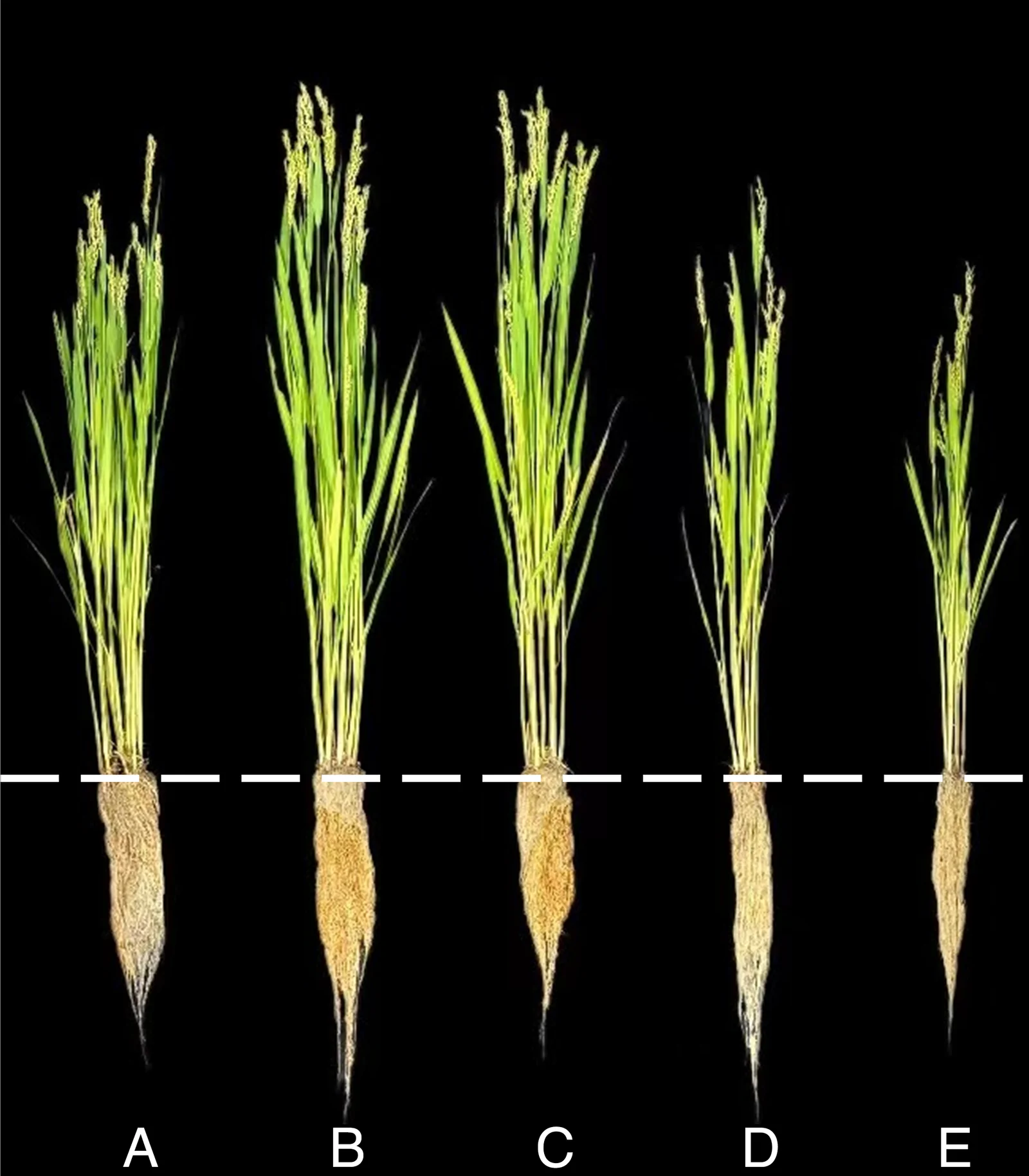
Growth phenotype of rice at maturity under different treatments. (**A**) NPK. (**B**) NPK + ZD-1. (**C**) ZD-1. (**D**) N + ZHS-1. (**E**) PK + GD2.

At the maturity stage, rice plants treated with ZD-1 alone (T3) exhibited significantly greater plant height, tiller number, and root biomass compared to the NPK control (T1; Fig. 8). Plants treated with NPK + ZD-1 (T2) showed similar growth parameters to T3, with slightly longer roots. No significant additive effect was observed when ZD-1 was combined with NPK fertilizer.

In the exploratory treatments, plants under N + ZHS-1 (T4) or PK + GD2 (T5) showed reduced plant height, fewer tillers, and thinner root systems compared to T1, T2, and T3. However, as these treatments lacked appropriate single-strain controls without fertilizer, they cannot be directly compared with T1–T3 to assess the standalone efficacy of the consortium vs individual strains. Therefore, the following statistical analyses and comparisons focus primarily on treatments 1, 2, and 3.

#### Nitrogen, phosphorus, and potassium accumulation in rice tissues

**Fig 9:**
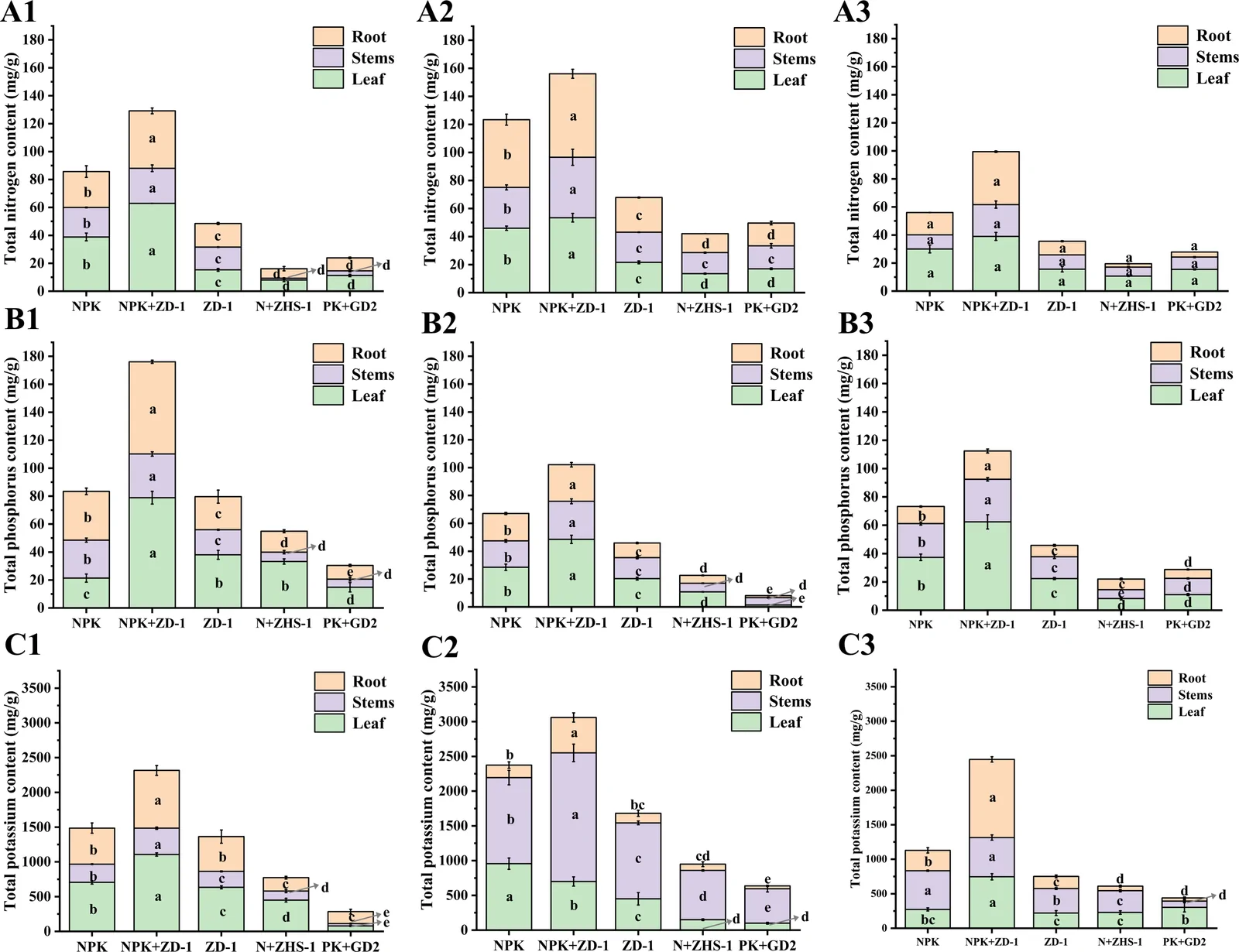
Nitrogen, phosphorus, and potassium accumulation in rice tissues. (**A1–A3**) Total nitrogen in roots, stems, and leaves at the seedling, filling, and maturity stages. (**B1–B3**) Total phosphorus. (**C1–C3**) Total potassium. Data are presented as mean ± SD (*n* = 5). Different letters indicate significant differences (*P* < 0.05).

Among treatments 1, 2, and 3, the NPK + ZD-1 treatment (T2) exhibited the highest overall nutrient accumulation. Total nitrogen (147.10 mg/g) and total potassium (3,059.95 mg/g) peaked at the grain filling stage, while total phosphorus (176.08 mg/plant) was highest at the seedling stage (Fig. 9). The ranking of nutrient content in these three treatments was consistent: T2 > T1 > T3 for N, P, and K across all growth stages, confirming that the use of ZD-1 (T2) significantly aids plants in better absorbing nutrients from the soil. Stage-specific characteristics were significant; the total nitrogen content was the highest at the grain filling stage (nitrogen is critical for yield and quality during this period), while the total phosphorus content was the highest at the seedling stage (37), which promoted root development (38). The total potassium content was the highest at the grain filling stage, which enhanced stem support and nutrient transport.

In the exploratory treatments (T4 and T5), the N + ZHS-1 group showed the lowest total nitrogen content among all treatments, while the PK + GD2 group exhibited the lowest total phosphorus and potassium contents. These observations suggest that single strains, when applied with partial fertilizer substitution, may not fully meet the plant’s nutritional demands under the tested conditions.

### Effects of ZD-1 on soil nutrient availability

**Fig 10:**
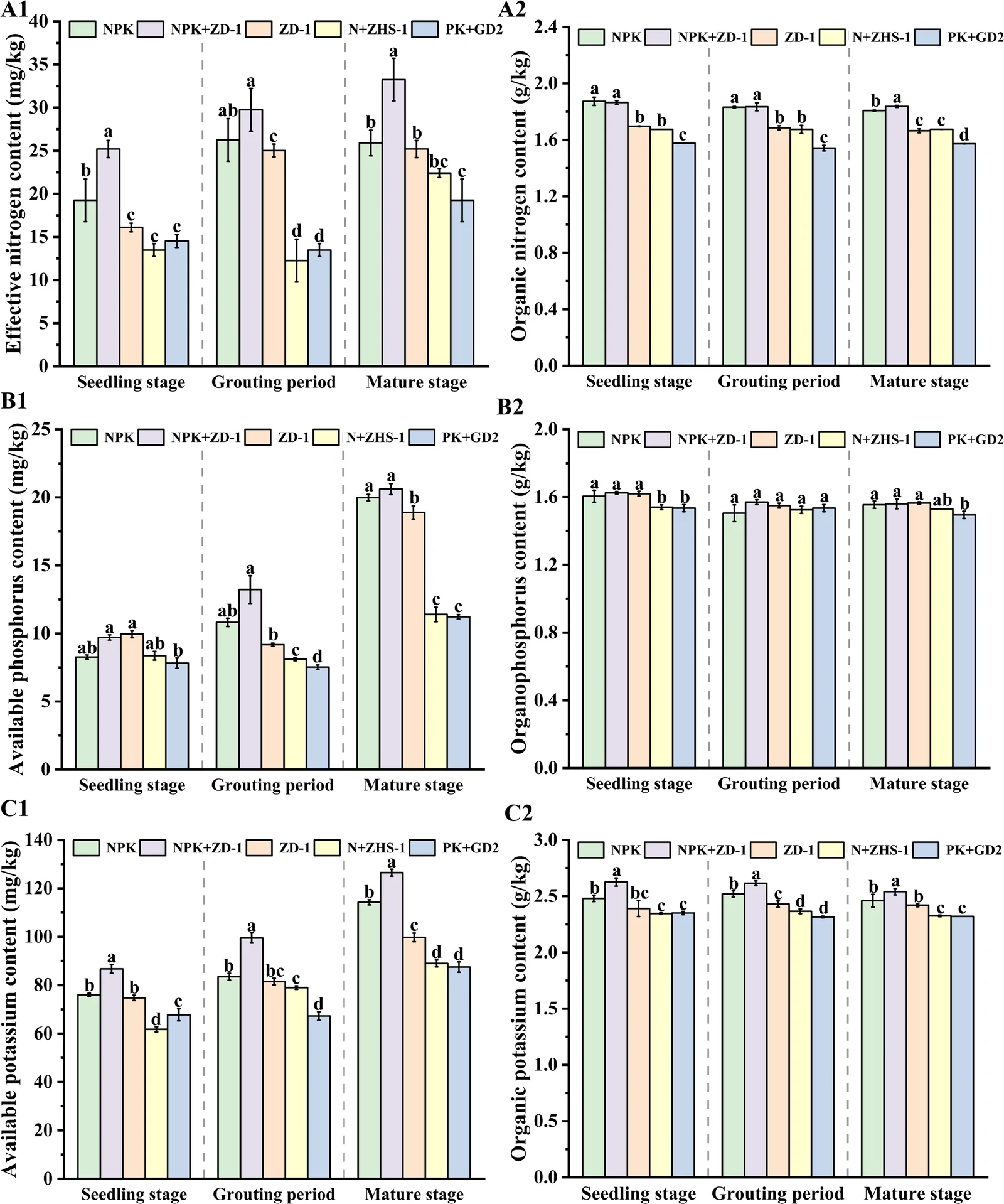
Soil nitrogen, phosphorus, and potassium contents under different treatments. (**A1 and A2**) Available nitrogen and total nitrogen. (**B1 and B2**) Available phosphorus and total phosphorus. (**C1 and C2**) Available potassium and total potassium. Data are presented as mean ± SD (*n* = 5). Different letters indicate significant differences (*P* < 0.05).

Across all five treatments, total nitrogen, phosphorus, and potassium contents in soil remained relatively stable from the seedling to maturity stage, whereas available nutrient contents showed significant variation (Fig. 10).

In the exploratory treatments, the soil available nitrogen content under PK + GD2 (T5) was comparable to that under N + ZHS-1 (T4) across all three growth stages, suggesting that GD2 possesses nitrogen-fixing capacity. Similarly, soil available phosphorus and potassium under N + ZHS-1 (T4) were slightly higher than those under PK + GD2 (T5) at the seedling and filling stages, with convergence by maturity, reflecting the phosphorus- and potassium-solubilizing capacity of ZHS-1.

Notably, among treatments 1, 2, and 3, the ZD-1 treatment (T3) significantly increased soil available nitrogen, phosphorus, and potassium compared to T1 (NPK alone), and its effects were comparable to those of T2 (NPK + ZD-1). This indicates that ZD-1 alone can effectively mobilize soil nutrients, achieving levels close to or exceeding those of chemical fertilization.

## DISCUSSION

Algal–bacterial consortium relies on synergistic interactions, but competitive and antagonistic interactions may also exist (39). Therefore, it is necessary to determine an appropriate ratio based on water quality to exert its benefits. Through analyses of growth traits and nutrient transformation capacities, this study identified the optimal ratio of the nitrogen-fixing cyanobacterium GD2 to the phosphate- and potassium-solubilizing bacterium ZHS-1, and further confirmed that ZHS-1 and GD2 can mutually promote each other’s growth and enhance their respective functional performances.

The algal–bacterial interaction follows a mutualistic pattern: algae synthesize organic carbon via photosynthesis, with a portion secreted as extracellular polysaccharides to serve as a carbon source for bacteria (40). When utilizing polysaccharides, bacteria release nitrogen-containing compounds; they can also transform nutrients and produce vitamins to promote algal growth (41). The chemotaxis of marine bacteria toward algal polysaccharides (42) can facilitate metabolic exchange and influence the carbon cycle (43). Algae synthesize proteins to support their own physiological activities (44); the organic substances they secrete are degraded by bacterial extracellular proteases and then utilized by bacteria (39). Additionally, co-cultivation can increase the yield of algal-secreted proteins (45, 46). Targeted analysis of GD2 and ZHS-1 revealed that the co-cultivation of ZHS-1 and GD2 led to a significant increase in EPSs, and various extracellular secretions from GD2 all exerted a chemotactic effect on ZHS-1, thereby facilitating the formation of biofilms from the extracellular substances of the two strains, which is the key to their non-antagonistic and mutually promoting growth.

Regarding the extracellular protein data presented in Fig. 4B, we acknowledge that extracellular proteins can originate from both active secretion and cell lysis. While increased biomass may lead to increased cell death and thus contribute to extracellular protein levels, our transcriptomic data provide additional insights into the metabolic state of the consortium. The upregulation of pheT (phenylalanyl-tRNA synthetase) in GD2 and ribosomal genes in both strains under co-culture conditions (see section “Transcriptomic analysis of synergistic interactions”) suggests enhanced protein synthesis capacity, which may support increased production and active secretion of extracellular proteins. This interpretation is consistent with the observed synergistic enhancement of biofilm formation (Fig. 4C) and extracellular polysaccharide production (Fig. 4A) in ZD-1, as these processes require active production and secretion of extracellular matrix components. Nevertheless, we acknowledge that the relative contributions of active secretion vs cell lysis to the extracellular protein pool cannot be definitively distinguished from the current data, and this represents a limitation of the present study.

Transcriptome analysis revealed the molecular mechanism underlying the formation of the algal–bacterial consortium: At the bacterial level, pathways such as 2-oxocarboxylic acid metabolism and oxidative phosphorylation, as well as ribosomal genes, are enriched (47, 48). This not only enhances respiratory ATP production to meet metabolic demands but also improves protein synthesis efficiency through the upregulation of ribosomal subunit proteins, supporting bacterial growth (49) and environmental adaptation (50). At the algal level, photosynthetic antenna proteins (e.g., carotenoids) enhance light energy capture and transfer (51). Photosynthesis converts CO₂ into organic substances to provide energy and participate in the carbon cycle (52), while carbon sequestration maintains the ecological carbon balance (53). Ribosomes synthesize proteins to support algal cell growth and division (54, 55), and phycocyanin fulfills both energy supply and nitrogen storage functions (56). ZHS-1 and GD2 exhibit significant synergistic effects. Their interaction promotes the growth of each other and the synthesis of metabolites, simultaneously enhancing the nitrogen-fixing capacity of GD2 and the phosphorus-solubilizing/k potassium-solubilizing capacity of ZHS-1. This optimizes resource utilization, comprehensively ensures the functional advantages of the consortium, and fully reflects the value of algal–bacterial interaction.

In the study investigating the feasibility of microbial inoculants replacing chemical fertilizers, we first conducted exploratory research on the application of single microbial inoculants: pot experiments were carried out by replacing chemical potassium fertilizer with the potassium-solubilizing bacterium ZHS-1 and chemical nitrogen fertilizer with the nitrogen-fixing cyanobacterium GD2, respectively. The results showed that the contents of available soil potassium/nitrogen and plant biomass in the two single inoculant treatment groups were significantly lower than those in the control group with direct chemical fertilizer application. This finding confirms the application limitations of single microbial inoculants in agricultural ecosystems, which stem from factors such as the limited nutrient transformation efficiency of single functional strains (56) and thus fail to meet the nutrient requirements of plants throughout their entire growth cycle. In stark contrast, the algae–bacteria composite inoculant ZD-1 not only significantly increased the contents of available soil nitrogen, phosphorus, and potassium, but also achieved a comprehensive nutrient supply capacity comparable to that of chemical fertilizers. Furthermore, the combined application of ZD-1 with chemical fertilizers exhibited an optimal synergistic effect: the soil nutrient pool (nitrogen, phosphorus, and potassium) was in a state of balanced and highly efficient utilization, and the plant biomass reached its peak across all treatments (55). This result not only elucidates the core mechanism by which the composite inoculant breaks through the application bottlenecks of single inoculants via inter-strain functional complementarity—specifically the synergistic effects of nitrogen fixation and carbon supply by GD2 and potassium and phosphorus solubilization by ZHS-1—but also provides direct experimental evidence for the construction of a green fertilization system characterized by “chemical fertilizer reduction coupled with efficiency enhancement of composite microbial inoculants” (57), which holds important practical significance for promoting the efficient nutrient utilization and sustainable development of agricultural ecosystems. We acknowledge that traditional plant growth-promoting traits such as auxin production and nitrogenase activity were not directly measured in the current study. While the nitrogen-fixing capability of GD2 and the IAA-producing ability of ZHS-1 have been previously characterized in our laboratory (19, 20), future studies should include direct measurements of these traits in the ZD-1 consortium to further elucidate the mechanistic basis of its growth-promoting effects.

## Supplementary Material

Reviewer comments

## Data Availability

Raw RNA-seq data have been deposited in the NCBI SRA database under BioProject PRJNA1450526.

## SUPPLEMENTAL MATERIAL

Fig. S1 spectrum.03506-25-s0001.docx Relative expression level of differentially expressed genes by RT-qPCR.

Table S1 spectrum.03506-25-s0002.docx Reverse transcription system.

Table S2 spectrum.03506-25-s0003.docx qPCR primer sequences.

Table S3 spectrum.03506-25-s0004.docx qPCR system.

Table S4 spectrum.03506-25-s0005.docx qPCR amplification condition.

OPEN PEER REVIEW reviewer-comments.pdf An accounting of the reviewer comments and feedback.

ASM does not own the copyrights to Supplemental Material that may be linked to, or accessed through, an article. The authors have granted ASM a non-exclusive, world-wide license to publish the Supplemental Material files. Please contact the corresponding author directly for reuse.
