# Supplementary material for: Study on the construction of algal–bacterial consortium comprising Pantoea vagans ZHS-1 and Nostoc sp. GD2 and its growth-promoting effect on rice
Source: Microbiol Spectr. 2026 Aug 31;14(10):e03506-25. doi: 10.1128/spectrum.03506-25 (PMC13688834; doi:10.1128/spectrum.03506-25)
Supplement: Supplementary file 1 [file reviewer-comments.pdf]

AMERICAN SOCIETY FOR MICROBIOLOGY | Microbiology Spectrum

Study on the Construction of Algal - Bacterial Consortium Comprising *Pantoea vagans* ZHS-1 and *Nostoc* sp. GD2 and Its Growth-Promoting Effect on Rice

Ji Dong, Chengcai Yue, Shiqi Tian, Xueying Zhou, Siyuan Li, Dongchao Wang, Zhihai Wu, Meiying Yang, Xue Yang, and Lei Wu

Corresponding Author(s): Lei Wu, Jilin Agricultural University

Review Timeline:

Submission Date:	November 26, 2025
Editorial Decision:	January 7, 2026
Revision Received:	March 11, 2026
Editorial Decision:	April 5, 2026
Revision Received:	April 9, 2026
Accepted:	April 30, 2026

Editor: Blaire Steven

Reviewer(s): Disclosure of reviewer identity is with reference to reviewer comments included in decision letter(s). The following individuals involved in review of your submission have agreed to reveal their identity: Paulo Ivan Fernandes Junior (Reviewer #1)

Transaction Report:

(Note: With the exception of the correction of typographical or spelling errors that could be a source of ambiguity, letters and reports are not edited. The original formatting of letters and referee reports may not be reflected in this compilation.)

DOI: https://doi.org/10.1128/spectrum.03506-25

Re: Spectrum03506-25 (**Study on the Construction of Algo - fungus Consortium and Its Growth - promoting Effect on Rice**)

Dear Dr. Lei Wu:

Thank you for the privilege of reviewing your work. Below you will find my comments, instructions from the Spectrum editorial office, and the reviewer comments.

After having read and received a review on the manuscript I am recommending modifications. Please see the review comments below.

Please return the manuscript within 60 days; if you cannot complete the modification within this time period, please contact me. If you do not wish to modify the manuscript and prefer to submit it to another journal, notify me immediately so that the manuscript may be formally withdrawn from consideration by Spectrum.

Revision Guidelines
To submit your modified manuscript, log into the submission site at https://spectrum.msubmit.net/cgi-bin/main.plex. Go to Author Tasks and click the appropriate manuscript title to begin. The information you entered when you first submitted the paper will be displayed; update this as necessary. Note the following requirements:

• Upload point-by-point responses to the issues raised by the reviewers in a file named "Response to Reviewers," NOT in your cover letter.
• Upload a compare copy of the manuscript (without figures) as a "Marked-Up Manuscript" file.
• Upload a clean .DOC/.DOCX version of the revised manuscript and remove the previous version.
• Each figure must be uploaded as a separate, editable, high-resolution file (TIFF or EPS preferred), and any multipanel figures must be assembled into one file.
• Any supplemental material intended for posting by ASM should be uploaded with their legends separate from the main manuscript. You can combine all supplemental material into one file (preferred) or split it into a maximum of 10 files with all associated legends included.

For complete guidelines on revision requirements, see our Submission and Review Process webpage. Submission of a paper that does not conform to guidelines may delay acceptance of your manuscript.

Data availability: ASM policy requires that data be available to the public upon online posting of the article, so please verify all links to sequence records, if present, and make sure that each number retrieves the full record of the data. If a new accession number is not linked or a link is broken, provide Spectrum production staff with the correct URL for the record. If the accession numbers for new data are not publicly accessible before the expected online posting of the article, publication may be delayed; please contact production staff (Spectrum@asmusa.org) immediately with the expected release date.

Publication Fees: For information on publication fees and which article types are subject to charges, visit ourwebsite. If your manuscript is accepted for publication and any fees apply, you will be contacted separately about payment during the production process; please follow the instructions in that e-mail. Arrangements for payment must be made before your article is published.

ASM Membership: Corresponding authors may join or renew ASM membership to obtain discounts on publication fees. Need to upgrade your membership level? Please contact Customer Service at Service@asmusa.org.

The ASM Journals program strives for constant improvement in our submission and publication process. Please tell us how we can improve your experience by taking this quick Author Survey.

Thank you for submitting your paper to Spectrum.

Sincerely,
Blaire Steven
Editor
Microbiology Spectrum

Reviewer #1 (Comments for the Author):

Dear Editor

The manuscript "Study on the Construction of Algo - fungus Consortium and Its Growth - promoting Effect on Rice" aimed to

assess a bacteria + algae consortium regarding several biochemical characteristics in BG11 culture medium, in addition to gene expression/validation assays and a plant growth-promotion evaluation.
Despite the idea and the potential of the data being good, several flaws in the manuscript must be addressed to deliver a more diligent report and broaden the paper´s audience.
My main concerns are methodological flaws or conceptual mistakes described in the next paragraphs.

Abstract and Importance:

The authors should include the scientific names of the bacteria and cyanobacterial strains.

Introduction:
The authors describe the overuse of chemicals in Chinese agriculture very well. However, the topic lacks information from other studies on algae and algae/bacteria inoculation in plants, showing positive results for plant growth and yield. After presenting these results, the authors would present previous results with ZHS-1 and GD2, pointing out that those previous studies with single inoculation (alongside the literature data) inspired the team to test them under coinoculation.

Materials and methods:
Subtopic 2.1: The authors must include the scientific names of both strains used in this manuscript. Instead of citing only their reference, the authors must include additional information on the previous characterization of both strains.

Subtopic 2.2.1: It was not clear why the authors tested the different proportions ZHS-1/GD2 in the culture medium. As I understood, proportions ranged from 1:1 to 1:5 (ZHS-1:GD2). Why didn't they test the opposite proportions too (from 1:1 to 5:1, ZHS-1:GD2)? This information must be in the test explained in the motivation for testing this range of proportions.

Subtopic 2.2.2: In lines 113-116, the authors argue that the measurement of N, K, and P in the supernatant indicated the N-fixation and the K and P solubilization potential of the consortia. However, they stated in the previous topic that after preparing the microbial mixes, they were "...inoculated into 100 mL of BG11 liquid medium to prepare the algae-bacteria seed suspension". Although the BG11 medium composition contains only 40 mg of K_2HPO_4 and no insoluble source of P and K, in this case, the micro-organisms have no insoluble P and K to dissolve. For N fixation, the ammonium in the medium is probably from the cell death and release, and not from the N fixation, since the fixed N will probably be used in full by the cyanobacterial filaments. The authors should reevaluate their argument for these measurements.

Subtopic 2.3.1: I was a little bit confused. So far, I know, the phenol-sulfuric acid method breaks down the EPS into its monosaccharides. For this reason, a standard curve of glucose can be used for quantification. The EPS is usually extracted with cold ethanol. Since the experiment has already been conducted, the authors should restate the aims and objectives of this analysis.

Subtopic 2.3.2: Since the bacterial and algae growth had already been determined by plate count and chlorophyll content analysis, respectively, why is the quantification of proteins important at this point?
Subtopic 2.5: How could the authors determine the molarity of GD-2 secretions?
The method the authors use to assess bacterial mobility in the solutions is interesting at first glance. But, I think that the different amounts of bacteria observed in the different BG11 media supplied with the different C sources could be due to the bacterial preference for these sources and, consequently, their multiplication ability inside each C medium. Before using this method for the chemotaxis approach, it must be validated. I think a good way to do this is to inoculate the ZHS-1 bacteria into the same syringes (without the tip containing bacteria) and check whether the OD or plate counts change after an incubation period. Changes in initial bacterial counts indicate bacterial growth, which is particularly notable in GD-2 exudates.

Subtopic 2.7.1: In the plant assay, I can see a complete experiment with three treatments: 1) NPK, 2) NPK + ZD-1, and 3) ZD-1. The comparisons among these treatments are fair, since they have the same idea and basis (consortia and/or fertilization). The other two treatments N+ZHS-1 and PK+GD2 were probably added to assess the viability of bactéria and algae as sources of PK and N, respectively. However, they do not have the proper controls, which should be the treatments PK, N, GD2, and ZHS-1. In this case, comparing these two spare treatments with the first three is not fair, and the results will not be sufficiently confident to reveal the real impact of the microbes on supplementing fertilization in rice. Despite these two treatments, the authors should have included the 50% NPK and 50% NPK + ZD-1 treatments to assess the ability of the microbial consortia to promote plant growth under limited fertilization conditions (if 50% NPK + ZD-1 = NPK, the result would be very good). As the experiment has already been done, I recommend analyzing, showing, and discussing only the three treatments cited above.

Subtopics 2.7.2 and 2.7.3: No methods were cited at all.

Results and Analysis:
Is the title of this section right?
Lack of statistical analysis for most of the biochemical or cell growth data in the manuscript. Proper statistics must be added and discussed.
Suggestion: traditional plant growth-promoting characteristics could be assessed in the single microbes and in the consortium.

Characteristics such as auxin production and nitrogenase activity (acethylene reduction assay) could add valuable data to this manuscript.

Figure 1: The plot is confused. The best way to plot and understand it is to have the bacteria and algae in the same plot. So they may be separated by the proportions. The figure title must be self-explanatory.

Subtopic 3.1.2: These results cannot be presented as N fixation and PK solubilization for the reasons explained above (Subtopic 2.2.2). After elaborating on the methodology and objectives of this analysis, the authors will need to restructure how they present these data.
Figure 2: The figure is poor and not readable.

Subtopic 3.2.1: As discussed above (Subtopic 2.5), I am not quite sure if this result is due to the bacterial migration from one matrix to another or if it is a result of bacterial growth in the new BG11 medium. Before a validation experiment for this new approach, this data is not trustworthy, and if the authors can not assess or validate it now, they should not use this data in this manuscript.

Subtopic 3.2.2: The intracellular and extracellular protein increase is expected, in my point of view. The extracellular protein content likely comes from cell death and lysis, and if there are more live cells, there should be more dead ones as well.
Lines 415-416: These assumptions are speculative, not the manuscript data.
Regarding the consortia's biofilm-forming capacity, a discussion of multispecies biofilms is worth noting, supported by literature data.

Subtopic 3.5.2: Could the nif genes upregulation in ZD-1 occur due to the higher algal growth in the medium? In this case, more cells and more gene expression compared to GD2 alone.
Figures 5, 6, and 7: The figures are not readable.

Subtopic 3.6, 3.7, and 3.8: As I pointed out in subtopic 2.7.1, the plant assay must be reanalyzed and the results presented again. After the new presentation, and in a more straightforward and assertive manner, this subtopic will be better aligned with the manuscript as a whole and will highlight the potential of bacterial consortia through fair analysis and comparisons.

Discussion

The discussion is vague. The authors cite several references that talk to each other but not to the manuscript's data. In this discussion, the authors must use the references to compare them with their data and the data's correlation.
In this section, the authors could correlate their data (among others): 1) optimization of ZD-1 consortium and the single strains behaviour in all different tested conditions; 2) growth (the authors call "chemotaxis") of ZHS-1 in GD2 exudates and biofilm formation; 3) consortium-growth and gene expression; 4) EPS production x biofilm formation x plant growth promotion. These results must "talk" to each other and use the references as support for your results.
After the restructuring of the data presentation, the changes in the discussion will be easier to make.

Response to Reviewers

Manuscript ID: Spectrum03506-25

Title: Study on the Construction of Algal-Bacterial Consortium Comprising *Pantoea vagans* ZHS-1 and *Nostoc* sp. GD2 and Its Growth-Promoting Effect on Rice

Dear Editor and Reviewers,

We would like to express our sincere gratitude to the editor and the reviewers for their valuable time and insightful comments on our manuscript. These comments have been extremely helpful in improving the quality and clarity of our work. We have carefully considered all the suggestions and have revised the manuscript accordingly. Below is our point-by-point response to each comment, along with a description of the changes made.

Reviewer #1

General Comment: The manuscript "Study on the Construction of Algo-fungus Consortium and Its Growth-promoting Effect on Rice" aimed to assess a bacteria + algae consortium regarding several biochemical characteristics in BG11 culture medium, in addition to gene expression/validation assays and a plant growth-promotion evaluation. Despite the idea and the potential of the data being good, several flaws in the manuscript must be addressed to deliver a more diligent report and broaden the paper's audience. My main concerns are methodological flaws or conceptual mistakes described in the next paragraphs.

Response: We sincerely thank the reviewer for the positive assessment of our study's potential and for the detailed and constructive comments. We have carefully addressed all the concerns raised and have revised the manuscript thoroughly to enhance its scientific rigor and clarity.

Abstract and Importance

Comment: The authors should include the scientific names of the bacteria and cyanobacterial strains.

Response: We agree with the reviewer. The scientific names of both strains have now been included in the Abstract, Importance and Keywords sections.

Changes in Manuscript:

- **Abstract (Lines 13-14):** "...comprising the potassium-solubilizing bacterium *Pantoea vagans* ZHS-1 and the nitrogen-fixing cyanobacterium *Desmonostoc muscorum* GD2."
- **Importance (Line 28-29):** "...composed of *Pantoea vagans* ZHS-1 and *Desmonostoc muscorum* GD2..."
- **Keywords (Line 36):** "**Keywords:** algal--bacterial consortium; *Pantoea vagans*; *Desmonostoc muscorum*; ..."

Introduction

Comment: The authors describe the overuse of chemicals in Chinese agriculture very well. However, the topic lacks information from other studies on algae and algae/bacteria inoculation in plants, showing positive results for plant growth and yield. After presenting these results, the authors would present previous results with ZHS-1 and GD2, pointing out that those previous studies with single inoculation (alongside the literature data) inspired the team to test them under coinoculation.

Response: We thank the reviewer for this valuable suggestion. We have expanded the Introduction

to include recent studies on algae-based fertilizers and algal-bacterial co-inoculation. We then present our previous findings with ZHS-1 and GD2, leading to the rationale for testing them in co-culture.

Changes in Manuscript (Introduction):

- Added references to Nguyen et al. (2025) and Yu et al. (2025) on algal fertilizers.
- Added a paragraph summarizing the previous characterization of ZHS-1 and GD2 (Lines 77-86).
- Revised the rationale to explicitly state that the limited effects of single strains in paddy soil inspired us to test them in a consortium (Lines 87-92).

Materials and Methods

2.1 Experimental strains and culture media

Comment: The authors must include the scientific names of both strains used in this manuscript. Instead of citing only their reference, the authors must include additional information on the previous characterization of both strains.

Response: We have revised this section to include the full scientific names and a brief summary of the key functional characteristics of each strain, as previously reported.

Changes in Manuscript (Lines 105-109):

"Strain ZHS-1 is elliptical rod-shaped and identified as *Pantoea vagans*, which possesses multiple functions of potassium solubilization, phosphate solubilization, and indole-3-acetic acid (IAA) secretion. Strain GD2 is a filamentous cyanobacterium identified as *Desmonostoc muscorum*, with a maximum nitrogenase activity of 75.33 U·mg^{-1} under optimal conditions, exhibiting a significant nitrogen-fixing function."

Scanning Electron Micrograph of ZHS-1 and GD2:

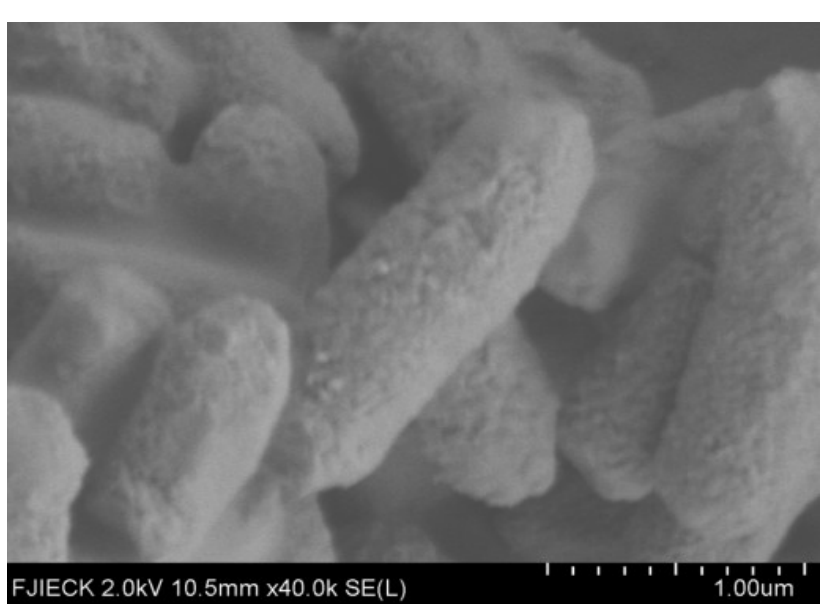

ZHS-1

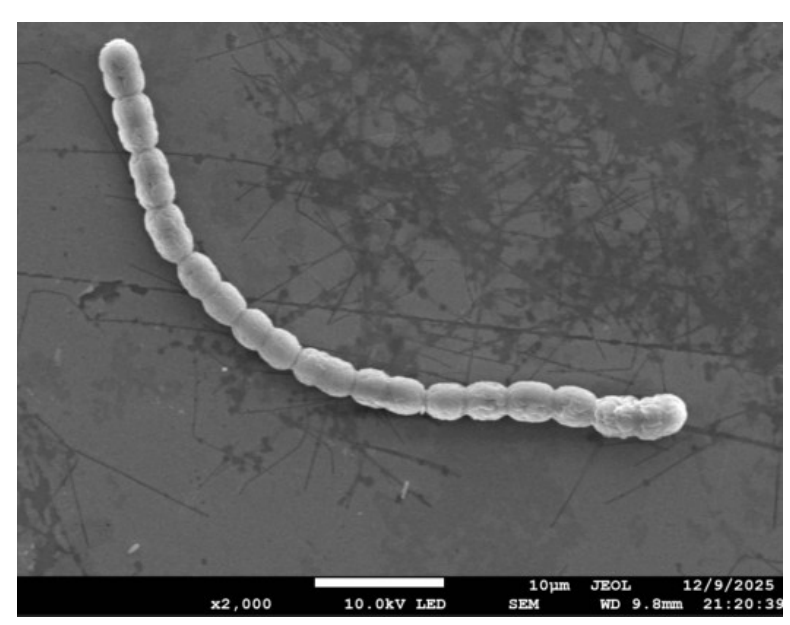

GD2

2.2.1 Algae-Bacteria co-cultivation experiment

Comment: It was not clear why the authors tested the different proportions ZHS-1/GD2 in the culture medium. As I understood, proportions ranged from 1:1 to 1:5 (ZHS-1:GD2). Why didn't they test the opposite proportions too (from 1:1 to 5:1, ZHS-1:GD2)? This information must be in the test explained in the motivation for testing this range of proportions.

Response: We thank the reviewer for this important question. We have now provided a clear scientific rationale for testing ratios with a higher proportion of GD2 relative to ZHS-1.

In algal-bacterial symbiotic systems, algal metabolites—particularly photosynthetically derived organic carbon—serve as the primary carbon source for heterotrophic bacteria. An excessively high proportion of bacteria could lead to intensified competition for limited nutrients, including nitrogen,

phosphorus, and algal-derived organic carbon. Such competition may result in nutrient deficiency and growth inhibition of the algae, potentially destabilizing the consortium. Therefore, to maintain a balanced and stable co-culture system, we focused on ratios where GD2 proportion is equal to or higher than that of ZHS-1.

Based on this rationale, five mass ratios (ZHS-1:GD2 = 1:1, 1:2, 1:3, 1:4, 1:5) were selected for systematic evaluation.

Changes in Manuscript (Lines 132-139):

"To evaluate the synergistic effects of ZHS-1 and GD2, five mass ratios (ZHS-1:GD2 = 1:1, 1:2, 1:3, 1:4, 1:5) were tested. The ratios were designed with a higher proportion of GD2 relative to ZHS-1, as algal metabolites typically serve as the primary carbon source for heterotrophic bacteria in algal-bacterial systems. An excessively high proportion of bacteria could lead to intensified competition for nutrients—such as nitrogen, phosphorus, and algal-derived organic carbon—potentially causing nutrient deficiency and growth inhibition of algae, and even disrupting the stability of the consortium (45, 46). The mixed cultures were inoculated into 100 mL BG11 liquid medium and incubated under the standard algal culture conditions for 7 days, with manual shaking every 12 h."

2.2.2 Screening for the optimal co-culture ratio

Comment: In lines 114-117, the authors argue that the measurement of N, K, and P in the supernatant indicated the N-fixation and the K and P solubilization potential of the consortia. However, they stated in the previous topic that after preparing the microbial mixes, they were "...inoculated into 100 mL of BG11 liquid medium to prepare the algae-bacteria seed suspension". Although the BG11 medium composition contains only 40 mg of K2HPO4 and no insoluble source of P and K, in this case, the micro-organisms have no insoluble P and K to dissolve. For N fixation, the ammonium in the medium is probably from the cell death and release, and not from the N fixation, since the fixed N will probably be used in full by the cyanobacterial filaments. The authors should reevaluate their argument for these measurements.

Response: We thank the reviewer for pointing out this conceptual issue. We have revised the description of these measurements to accurately reflect what they represent: the net release of nutrients into the medium, which results from combined processes including nutrient mobilization, metabolite release, and cellular turnover.

Regarding the nitrogen content determination, we agree with the reviewer that BG11 medium contains $NaNO_3$ as a nitrogen source, and that nitrogen accumulation in the supernatant may partially originate from cell death and release. Therefore, in the revised manuscript, we have clearly stated (Lines 156-158) that the mixtures were inoculated into BG11 liquid medium, and we noted that the observed nitrogen accumulation cannot be directly attributed to biological nitrogen fixation; it likely results from combined processes including metabolite release and cellular turnover.

Regarding the determination of phosphate-solubilizing capacity, we clearly indicated in the original manuscript (Line 170) that a **liquid inorganic phosphorus medium** was used, and its formulation was provided in **Section 2.2.1 Experimental Strains and Culture Media** (Lines 112–114). This medium contains insoluble tricalcium phosphate ($Ca_3(PO_4)_2$) as a phosphorus source, making it suitable for evaluating the phosphate-solubilizing ability of microorganisms.

Regarding the determination of potassium-solubilizing capacity, we clearly indicated in the original manuscript (Line 184) that a **modified potassium-solubilizing medium** was used, and its

formulation was provided in **Section 2.2.1 Experimental Strains and Culture Media** (Lines 116–120). This medium contains insoluble potassium feldspar powder as a potassium source, making it suitable for evaluating the potassium-solubilizing ability of microorganisms.

Therefore, the determinations of phosphate- and potassium-solubilizing capacities were conducted in specific media containing insoluble P/K sources, not in BG11 medium. We have clearly distinguished the different media used for each determination in the revised manuscript to avoid any confusion.

Changes in Manuscript:

- **Lines 152-156:** " These measurements reflect the net release of nutrients into the medium under different culture conditions. For phosphorus and potassium analyses, the media contained insoluble P/K sources, allowing assessment of solubilization capacity. For nitrogen analysis in BG11 medium, the measured accumulation results from combined processes including metabolite release and cellular turnover."
- **Lines 157-158:** "For nitrogen content analysis, the mixtures were inoculated into BG11 liquid medium..."
- **Line 171:** Clearly states that the phosphate-solubilizing capacity determination used liquid inorganic phosphorus medium.
- **Line 184:** Clearly states that the potassium-solubilizing capacity determination used modified potassium-solubilizing medium.
- **Results Section 3.1.2, Lines 411-413:** "Because BG11 medium contains a high concentration of $NaNO_3$, the observed nitrogen accumulation cannot be directly attributed to biological nitrogen fixation; it likely results from combined processes including metabolite release and cellular turnover."

2.3.1 Extracellular Polysaccharide Quantification

Comment: I was a little bit confused. So far, I know, the phenol-sulfuric acid method breaks down the EPS into its monosaccharides. For this reason, a standard curve of glucose can be used for quantification. The EPS is usually extracted with cold ethanol. Since the experiment has already been conducted, the authors should restate the aims and objectives of this analysis.

Response: We thank the reviewer for the opportunity to clarify. The aim of this analysis was to evaluate the potential of ZD-1 in biofilm formation and intercellular adhesion by quantifying the major components of EPS—extracellular polysaccharides and extracellular proteins. As EPS is primarily composed of these substances, their quantification provides a reliable assessment of EPS production, which is closely associated with biofilm formation and cell-cell interactions in algal-bacterial consortia.

Changes in Manuscript (Lines 191-192):

"To evaluate the potential of ZD-1 in biofilm formation and intercellular adhesion, extracellular polysaccharides—a major component of extracellular polymeric substances (EPS)—were quantified. "

2.3.2 Determination of Extracellular Protein Contents

Comment: Since the bacterial and algae growth had already been determined by plate count and chlorophyll content analysis, respectively, why is the quantification of proteins important at this point?

Response: We appreciate the reviewer's question. While plate counts and chlorophyll content provide information on biomass, they do not directly reflect metabolic activity or the production of functional extracellular compounds that mediate algal-bacterial interactions. Extracellular proteins, as key components of EPS, play essential roles in biofilm formation, cell adhesion, and intercellular communication. Therefore, quantifying extracellular protein content offers valuable insights into the physiological state of the consortium and the mechanisms underlying their synergistic interaction.

Accordingly, we have retained the determination of extracellular proteins in this study. In contrast, the analysis of intracellular protein content, which was considered redundant for the objectives of this work, has been completely removed from the manuscript to maintain focus and clarity.

Changes in Manuscript:

- A brief explanation of the rationale for extracellular protein quantification has been added at the beginning of section 2.3.2.
- All content related to intracellular protein determination has been deleted from the Materials and Methods, Results, and Figures sections.

2.3.4 Chemotaxis Assay

Comment 1: How could the authors determine the molarity of GD-2 secretions?

Response: The reviewer is correct; it is not possible to determine the molarity of a complex mixture like GD2 culture filtrate. We have corrected this terminology in the manuscript.

Changes in Manuscript (Lines 234-236):

"Sterile-filtered GD2 culture filtrate, as well as 1 M solutions of glucose, sucrose, maltose, trehalose, rhamnose, mannose, mannitol, and betaine prepared in BG11 liquid medium, were used as chemotactic solutions."

Comment 2: The method the authors use to assess bacterial mobility in the solutions is interesting at first glance. But, I think that the different amounts of bacteria observed in the different BG11 media supplied with the different C sources could be due to the bacterial preference for these sources and, consequently, their multiplication ability inside each C medium. Before using this method for the chemotaxis approach, it must be validated. I think a good way to do this is to inoculate the ZHS-1 bacteria into the same syringes (without the tip containing bacteria) and check whether the OD or plate counts change after an incubation period. Changes in initial bacterial counts indicate bacterial growth, which is particularly notable in GD-2 exudates.

Response: We thank the reviewer for this insightful comment, which touches upon a fundamental question in chemotaxis research: distinguishing between active migration and in situ proliferation.

We acknowledge that without direct growth controls, the observed differences in CFU could theoretically arise from differential bacterial multiplication within the test solutions. However, several lines of evidence support our interpretation that the observed RCI values primarily reflect chemotactic migration rather than differential growth:

First, if the RCI values were predominantly determined by bacterial growth rates in the test solutions, we would expect a positive correlation between the metabolic availability of the carbon source and the resulting CFU counts. Simpler sugars that are more readily metabolized should support faster bacterial growth and thus yield higher RCI values. However, our data show the opposite pattern: trehalose—a disaccharide that is typically metabolized more slowly than monosaccharides such as glucose—exhibited the highest RCI value (5.2), significantly exceeding

that of glucose (3.6). This inverse relationship between expected metabolic rate and observed bacterial accumulation strongly suggests that the differential CFU counts are driven by chemotactic attraction rather than differential growth.

Second, the RCI value for GD2 culture filtrate (4.7) fell between those of trehalose and rhamnose, which is consistent with a complex mixture containing multiple chemoattractants. If bacterial growth were the dominant factor, we would expect the complex filtrate to support intermediate growth rates—which it does—but this alone cannot explain the specific ranking of attractants that aligns with known chemotactic preferences of related bacteria reported in the literature [1-3].

Third, the capillary assay format itself limits the potential for substantial in situ growth during the 120-minute incubation period. Previous studies have demonstrated that under these conditions, the contribution of bacterial proliferation to the observed CFU differences is negligible compared to chemotactic accumulation [4,5].

We agree with the reviewer that direct growth controls would provide even stronger evidence. While such experiments were not included in the current study, we have now acknowledged this limitation in the revised Discussion section and propose that future investigations could include growth curves of ZHS-1 in each test solution to definitively separate chemotaxis from growth effects. Nevertheless, based on the reasoning above and the consistency of our results with established chemotaxis literature [1-5], we are confident that the observed RCI values reliably reflect chemotactic responses.

References Cited in This Response:

1. Adler J. A method for measuring chemotaxis and use of the method to determine optimum conditions for chemotaxis by Escherichia coli. Journal of General Microbiology. 1973;74(1):77-91.

2. Lewus P, Ford RM. Quantification of random motility and chemotaxis bacterial transport coefficients using individual-cell and population-scale assays. Biotechnology and Bioengineering. 2001; 75(3):292-304.

3. Ford RM, Lauffenburger DA. A simple expression for quantifying bacterial chemotaxis using capillary assay data: application to the analysis of enhanced chemotactic responses from growth-limited cultures. Mathematical Biosciences. 1992; 109(2):127-149.

4. Park H, Guet CC, Emonet T, Cluzel P. Fine-tuning of chemotactic response in E. coli determined by high-throughput capillary assay. Current Biology. 2011; 21(9):764-769.

5. Li J, Go AC, Ward MJ, Ottemann KM. The chemical-in-plug bacterial chemotaxis assay is prone to false positive responses. BMC Research Notes. 2010; 3:77.

2.5.1 Experimental Design and Treatments

Comment: In the plant assay, I can see a complete experiment with three treatments: 1) NPK, 2) NPK + ZD-1, and 3) ZD-1. The comparisons among these treatments are fair, since they have the same idea and basis (consortia and/or fertilization). The other two treatments N+ZHS-1 and PK+GD2 were probably added to assess the viability of bacteria and algae as sources of PK and N, respectively. However, they do not have the proper controls, which should be the treatments PK, N, GD2, and ZHS-1. In this case, comparing these two spare treatments with the first three is not fair, and the results will not be sufficiently confident to reveal the real impact of the microbes on supplementing fertilization in rice. Despite these two treatments, the authors should have included the 50% NPK and 50% NPK + ZD-1 treatments to assess the ability of the microbial consortia to

promote plant growth under limited fertilization conditions (if 50% NPK + ZD-1 = NPK, the result would be very good). As the experiment has already been done, I recommend analyzing, showing, and discussing only the three treatments cited above.

Response: We fully agree with the reviewer's assessment. We have followed the recommendation to focus our main analysis and conclusions on Treatments 1, 2, and 3. Treatments 4 and 5 are now explicitly described as exploratory groups in both the Methods and Results sections, and we have clearly stated that they cannot be directly compared with the other treatments due to the lack of proper controls. Their data are presented for transparency but are not used for statistical comparison or to draw main conclusions.

Changes in Manuscript:

- **Methods Section 2.5.1, Lines 270-282:** Added a paragraph explicitly stating the exploratory nature of T4 and T5 and the limitation in comparing them with T1-T3.
- **Results Section 3.4.1, Lines 542-545:** Added a similar caveat before presenting the T4/T5 data.
- **Results Sections 3.4.2 and 3.5:** The primary statistical comparisons and conclusions are now drawn from T1, T2, and T3.
- **Discussion Section, Lines 677-682:** Added a paragraph acknowledging this experimental design limitation.

2.7.2 and 2.7.3

Comment: No methods were cited at all.

Response: We have added the appropriate citations for the plant and soil nutrient determination methods.

Changes in Manuscript:

- **Line 344:** Added citation (27) for the nitrogen distillation method.
- **Line 457:** Added citation (28) for the phosphorus colorimetric method.
- **Line 461:** Added citation (29) for the soil available nitrogen method.
- **Line 494:** Added citation (30) for the soil total nitrogen method.

Results and Analysis

Comment 1: Is the title of this section right?

Response: We thank the reviewer for this important question. The reviewer is correct to question the title, and we have carefully reconsidered both the main section title and all subsection titles within the Results to ensure they accurately reflect the data presented.

First, regarding the main section title: We have changed it from "Results and Analysis" to the standard "RESULTS" as per journal guidelines (Line 385). This modification aligns with the formatting requirements of *Microbiology Spectrum* and is more consistent with standard scientific reporting conventions.

Second, and more importantly, we have thoroughly reviewed all subsection titles to ensure they accurately reflect the data presented and avoid any over-interpretation. In particular:

- Section 3.1.2: The original title "Nitrogen Fixation, Phosphorus Solubilization, and Potassium Solubilization by the Bacteria-Cyanobacteria Consortium" has been revised to "Nutrient Turnover Capacity of the Consortium" (Line 403). This change addresses the reviewer's valid concern (raised in Subtopic 2.2.2) that measurements in BG11 medium

cannot be directly attributed to biological nitrogen fixation, and now more accurately describes the net nutrient release observed in the supernatants.

- Section 3.2.2: The title has been revised to "Extracellular Polysaccharide, Extracellular Protein Production and Biofilm Formation" (Line 454) to clearly indicate which parameters were measured. The original title was imprecise and has been corrected to reflect that we quantified extracellular polysaccharides and extracellular proteins—major components of EPS—rather than total EPS.
- All other subsection titles have been checked for accuracy and consistency with the data presented, and any ambiguous or overstated terms have been revised accordingly.

These revisions ensure that the Results section, including all titles, accurately and transparently communicates our findings without overstatement.

Comment 2: Lack of statistical analysis for most of the biochemical or cell growth data in the manuscript. Proper statistics must be added and discussed.

Response: We thank the reviewer for pointing out this important omission. We have now performed statistical analyses for the quantitative data presented in the manuscript and have made corresponding revisions to the relevant figures and text.

Specifically:

- **Statistical analyses:** One-way analysis of variance (ANOVA) followed by Duncan's multiple range test was applied to all quantitative data, including growth curves (ZHS-1 CFU counts and GD2 chlorophyll content in **Fig. 1**), nutrient turnover measurements (nitrogen, phosphorus, and potassium concentrations in **Fig. 2**), EPS-related parameters (extracellular polysaccharide and extracellular protein contents in **Fig. 4A, B**), biofilm formation (OD_{590} values in **Fig. 4C**), plant nitrogen, phosphorus, and potassium accumulation (**Fig. 9**), and soil available nutrient contents (**Fig. 10**).
- **Significance indicators:** Different letters indicating statistically significant differences ($P < 0.05$) have been added to **Figures 1, 2, 4, 9, and 10**. These letters allow readers to readily identify significant differences between treatments or time points.
- **Figure legends:** The legends for all figures have been updated to explicitly state that data are presented as mean ± standard deviation (SD) and that different letters indicate significant differences at $P < 0.05$.
- **Results text:** The Results section has been revised to include appropriate references to the statistical findings. For example:
- In Section 3.1.1, we now state: "**on day 3, the colony count of ZHS-1 reached 26.67 × 10^5 CFU/mL, which was significantly higher than that of other ratios ($P < 0.05$; Fig. 1A).** "
- In Section 3.1.2, we now state: "**the 1:1 consortium displayed significantly higher soluble P than the ZHS-1 control from day 3 onward ($P < 0.05$; Fig. 2B).** "
- In Section 3.2.2, we now state: "**The biofilm-forming capacity of ZD-1 was significantly superior to that of single strains, with OD_{590} values 4.11-fold and 15.14-fold higher than those of ZHS-1 and GD2, respectively ($P < 0.05$; Fig. 4C).** "
- In Section 3.4.2, we now state: "**Among Treatments 1, 2, and 3, the NPK + ZD-1 treatment (T2) exhibited the highest overall nutrient accumulation, with significant differences compared to T1 and T3 ($P < 0.05$; Fig. 9).** "
- In Section 3.5, we now state: "**Notably, among Treatments 1, 2, and 3, the ZD-1**

treatment (T3) significantly increased soil available nitrogen, phosphorus, and potassium compared to T1 (NPK alone) ($P < 0.05$; Fig. 10). "

These additions ensure that the key conclusions drawn from the biochemical and plant growth data are statistically robust and properly discussed, addressing the reviewer's concern about the lack of statistical support in the original manuscript.

Suggestion: Traditional plant growth-promoting characteristics could be assessed in the single microbes and in the consortium. Characteristics such as auxin production and nitrogenase activity (acetylene reduction assay) could add valuable data to this manuscript.

Response: We thank the reviewer for this valuable suggestion. We agree that assessing traditional plant growth-promoting traits such as auxin production and nitrogenase activity would provide additional insights into the mechanisms underlying the beneficial effects of ZD-1.

However, the current study was designed to focus on the synergistic interactions between ZHS-1 and GD2 at the physiological, biochemical, and transcriptomic levels, with an emphasis on nutrient mobilization (nitrogen, phosphorus, and potassium) and plant growth promotion. While auxin production and nitrogenase activity are indeed important characteristics, they were beyond the scope of the present investigation.

Notably, the nitrogen-fixing capability of GD2 has been previously characterized in our laboratory, with a maximum nitrogenase activity of 75.33 $U \cdot mg^{-1}$ under optimal conditions, as reported in our earlier work (Wu et al., 2025). For ZHS-1, our previous studies have demonstrated its ability to secrete indole-3-acetic acid (IAA) (Tian et al., 2024).

We appreciate this suggestion and will consider incorporating these traditional plant growth-promoting assays in future studies to further elucidate the mechanisms of action of the ZD-1 consortium. We have now added a sentence in the Discussion section acknowledging this as a direction for future research (Lines 699-700).

Changes in Manuscript:

- **Discussion Section, Lines 699-700:** Added a sentence acknowledging that direct measurements of auxin production and nitrogenase activity in the ZD-1 consortium should be included in future studies to further elucidate the mechanistic basis of its growth-promoting effects.

Figure 1

Comment: The plot is confused. The best way to plot and understand it is to have the bacteria and algae in the same plot. So they may be separated by the proportions. The figure title must be self-explanatory.

Response: We thank the reviewer for this valuable suggestion regarding the presentation of Figure 1.

We understand the reviewer's recommendation to merge the two separate panels into a single integrated plot showing both bacterial and algal data together. However, after careful consideration, we have chosen to retain the current format with two separate panels (A and B) within the same figure, as we believe this presentation allows readers to clearly visualize the growth dynamics of each strain individually while still enabling easy comparison between different co-culture ratios.

To address the reviewer's concern about clarity, we have revised the figure title and legends to be more self-explanatory. The title now clearly states: "The growth of ZHS-1 and GD2 under different co-culture ratios." The legends have been updated to accurately describe each panel: "(A)

Colony-forming units (CFU) of ZHS-1; (B) Chlorophyll a content of GD2."

These revisions ensure that readers can clearly understand what each panel represents without ambiguity. We believe this addresses the reviewer's concern regarding the clarity of the figure title and legends, even while retaining the original panel format.

Changes in Manuscript (Figure 1, Lines 400-402):

"**Fig.1 The growth of ZHS-1 and GD2 under different co-culture ratios.** (A) Colony-forming units (CFU) of ZHS-1. (B) Chlorophyll a content of GD2."

We would like to express our sincere gratitude to the reviewer again for your careful observation and meticulous scrutiny, which helped us improve the clarity of our data presentation.

Subtopic 3.1.2 and Figure 2

Comment: These results cannot be presented as N fixation and PK solubilization for the reasons explained above (Subtopic 2.2.2). After elaborating on the methodology and objectives of this analysis, the authors will need to restructure how they present these data. Figure 2: The figure is poor and not readable.

Response: We have restructured the presentation of these data. The section title has been changed to "Nutrient Turnover Capacity," and the text has been revised to describe the observations without over-interpreting them as direct measures of fixation/solubilization. We have also generated a new, high-resolution version of Figure 2 with improved readability.

Changes in Manuscript:

- **Section Title, Line 403: "3.1.2 Nutrient Turnover Capacity of the Consortium"**
- **Lines 404-406:** "To evaluate nutrient transformation-related parameters in co-cultures with different ratios, the contents of total nitrogen (in BG11 medium), soluble phosphorus (in IPM) and soluble potassium (in MKSM) were monitored over 7 days."
- **Figure 2 (Line 426):** Replaced with a high-resolution, clearly labeled figure.

Subtopic 3.2.1 (Chemotaxis)

Comment: As discussed above (Subtopic 2.5), I am not quite sure if this result is due to the bacterial migration from one matrix to another or if it is a result of bacterial growth in the new BG11 medium. Before a validation experiment for this new approach, this data is not trustworthy, and if the authors can not assess or validate it now, they should not use this data in this manuscript.

Response: We thank the reviewer for this comment. As detailed in our response to the methodological concerns raised in Subtopic 2.3.4, we have provided multiple lines of evidence supporting the reliability of our chemotaxis data, including the inverse relationship between expected metabolic rates and observed RCI values, as well as supporting literature demonstrating the validity of this approach [1-5]. Based on these considerations, we are confident that the data presented in Fig. 3 reliably reflect genuine chemotactic responses and should be retained in the manuscript.

Subtopic 3.2.2

Comment 1: The intracellular and extracellular protein increase is expected, in my point of view. The extracellular protein content likely comes from cell death and lysis, and if there are more live cells, there should be more dead ones as well.

Response: We thank the reviewer for this insightful comment. We agree that extracellular proteins

can originate from cell death and lysis. However, in this study, we focused specifically on extracellular protein content as an indicator of metabolic activity and the production of functional extracellular compounds that mediate algal-bacterial interactions, rather than as a measure of total protein synthesis.

It is important to clarify that intracellular protein content was not determined in this study. As described in our response to the methodological comments in Section 2.3.2, all content related to intracellular protein measurement has been removed from the manuscript to maintain focus and clarity. The extracellular protein data presented in Fig. 4B reflect the proteins actively secreted or released into the culture supernatant by ZHS-1, GD2, and the ZD-1 consortium.

While we acknowledge that cell lysis may contribute to extracellular protein levels, our transcriptomic data provide additional insights. The upregulation of *pheT* (phenylalanyl-tRNA synthetase) in GD2 and ribosomal genes in both strains under co-culture conditions (Section 3.3) suggests enhanced protein synthesis capacity, which may support increased production and active secretion of extracellular proteins. This interpretation is consistent with the observed synergistic enhancement of biofilm formation (Fig. 4C) and EPS production (Fig. 4A) in ZD-1.

We have revised the Results section to present the extracellular protein data without over-speculation, and we have incorporated this nuanced discussion of the potential sources and functional significance of extracellular proteins in the revised Discussion section.

Comment 2: Lines 416-417: These assumptions are speculative, not the manuscript data.

Response: We have removed the speculative statements from the Results section.

Changes in Manuscript (Lines 468-470):

The original speculative sentences have been removed. The text now simply reports the findings: "The extracellular polysaccharide contents... increased with growth... The biofilm-forming capacity of ZD-1 was significantly superior..."

Comment 3: Regarding the consortia's biofilm-forming capacity, a discussion of multispecies biofilms is worth noting, supported by literature data.

Response: We have expanded the discussion of biofilm formation in the Discussion section, incorporating literature on multispecies biofilms.

Changes in Manuscript (Discussion, Lines 648-650):

"...thereby facilitating the formation of biofilms from the extracellular substances of the two strains, which is the key to their non-antagonistic and mutually promoting growth (59)."

Subtopic 3.5.2

Comment: Could the nif genes upregulation in ZD-1 occur due to the higher algal growth in the medium? In this case, more cells and more gene expression compared to GD2 alone.

Response: This is a valid consideration. The reviewer is correct that increased biomass could contribute to higher overall gene expression. However, our RT-qPCR data were normalized to reference genes and represent *relative expression levels per cell*. Therefore, the observed upregulation of *nif* genes in ZD-1 compared to GD2 alone indicates a true increase in transcriptional activity per cell, not just a biomass effect. We have added a brief clarification in the text to address this point.

Changes in Manuscript (Lines 537-540):

"The upregulation of these genes, as determined by RT-qPCR with normalization to reference genes, indicates a true increase in transcriptional activity per cell in the co-culture context, rather than being

solely a consequence of increased biomass."

Figures 5, 6, and 7

Comment: The figures are not readable.

Response: We have generated new, high-resolution versions of Figures 5, 6, and 7. The resolution, font sizes, and labels have been optimized for clarity.

Subtopic 3.6, 3.7, and 3.8 (Plant Data)

Comment: As I pointed out in subtopic 2.7.1, the plant assay must be reanalyzed and the results presented again. After the new presentation, and in a more straightforward and assertive manner, this subtopic will be better aligned with the manuscript as a whole and will highlight the potential of bacterial consortia through fair analysis and comparisons.

Response: As detailed in our response to the comment on 2.7.1, we have restructured the presentation of the plant data. The primary analysis and conclusions are now focused on Treatments 1, 2, and 3. The data from Treatments 4 and 5 are presented as exploratory observations with appropriate caveats.

Discussion

Comment: The discussion is vague. The authors cite several references that talk to each other but not to the manuscript's data. In this discussion, the authors must use the references to compare them with their data and the data's correlation. In this section, the authors could correlate their data (among others): 1) optimization of ZD-1 consortium and the single strains behaviour in all different tested conditions; 2) growth (the authors call "chemotaxis") of ZHS-1 in GD2 exudates and biofilm formation; 3) consortium-growth and gene expression; 4) EPS production x biofilm formation x plant growth promotion. These results must "talk" to each other and use the references as support for your results. After the restructuring of the data presentation, the changes in the discussion will be easier to make.

Response: We thank the reviewer for this insightful comment, which has guided us to thoroughly restructure the Discussion section to ensure that all claims are firmly grounded in our data and supported by relevant literature.

We have now revised the Discussion section to establish clear connections between our key findings and the existing literature, following the four areas suggested by the reviewer:

1. Consortium optimization and single-strain behavior: In the first paragraph, we discuss how the optimal 1:1 ratio, identified through growth dynamics (Fig. 1) and nutrient turnover data (Fig. 2), reflects a balance between mutualism and competition. This finding is contextualized within the framework of algal-bacterial interactions, supported by literature on metabolic complementarity and nutrient exchange (e.g., Ramanan et al., 2016; Matthews et al., 2023).

2. Chemotaxis and biofilm formation: In the second paragraph, we directly link the strong chemotactic response of ZHS-1 to GD2 exudates (RCI = 4.7, Fig. 3) with the enhanced extracellular polysaccharide production and biofilm formation in ZD-1 (Fig. 4). This connection is supported by studies demonstrating that chemotaxis-mediated attraction facilitates initial colonization and subsequent biofilm development in algal-bacterial systems (e.g., Raina et al., 2023; Clerc et al., 2023).

3. Consortium growth and gene expression: In the third paragraph, we correlate the

transcriptomic data (Fig. 5-7) with the observed phenotypic outcomes. Specifically, we link the upregulation of *galF* in ZHS-1 to increased extracellular polysaccharide production (Fig. 4A), and the upregulation of *nifH*, *nifD*, and *nifV* in GD2 to enhanced nitrogen availability (Fig. 2A, 9A). The upregulation of ribosomal genes and *pheT* is discussed in the context of enhanced protein synthesis capacity, which aligns with the increased extracellular protein content observed in ZD-1 (Fig. 4B). The RT-qPCR data normalized to reference genes confirm that these changes reflect true increases in transcriptional activity per cell rather than merely increased biomass.

4. EPS production, biofilm formation, and plant growth promotion: Throughout the discussion, we synthesize these findings to propose a mechanistic model wherein chemotaxis-driven recruitment leads to robust biofilm formation, creating a protective and efficient microenvironment that facilitates nutrient exchange and metabolic synergy. This ultimately results in enhanced plant growth and soil nutrient activation (Fig. 8-10). This model is supported by literature on the role of biofilms in plant-microbe interactions (e.g., Zhang et al., 2020; Balducci et al., 2023).

Additionally, we have added a paragraph at the end of the Discussion acknowledging the limitations of this study, including the absence of direct measurements of traditional plant growth-promoting traits such as auxin production and nitrogenase activity, while noting that these have been previously characterized in our laboratory (Tian et al., 2024; Wu et al., 2025) and should be addressed in future studies. We also acknowledge the limitation regarding the potential contribution of cell lysis to extracellular protein levels, while presenting transcriptomic evidence supporting active secretion.

These revisions ensure that the Discussion section now provides a coherent narrative that integrates all major findings, demonstrates how our data "talk" to each other, and is firmly supported by relevant literature.

We hope that these revisions meet the approval of the editor and reviewers. We are grateful for the opportunity to improve our manuscript and believe it is now significantly stronger.

Sincerely,

The Authors

Re: Spectrum03506-25R1 (**Study on the Construction of Algal - Bacterial Consortium Comprising *Pantoea vagans* ZHS-1 and *Nostoc* sp. GD2 and Its Growth-Promoting Effect on Rice**)

Dear Dr. Lei Wu:

Thank you for the privilege of reviewing your work. Below you will find my comments, instructions from the Spectrum editorial office, and the reviewer comments.

After having read the manuscript and response to reviewers comments I think the authors have addressed the reviewers concerns and I do not have ant remaining concerns with the quality of the science. However, the journal requires a dedicated data availability statement prior to publication. Please make sure this is included, and that all data that underlies the conclusions (particularly the transcriptomic datasets) are made publicly available

Please return the manuscript within 60 days; if you cannot complete the modification within this time period, please contact me. If you do not wish to modify the manuscript and prefer to submit it to another journal, notify me immediately so that the manuscript may be formally withdrawn from consideration by Spectrum.

Revision Guidelines
To submit your modified manuscript, log into the submission site at https://spectrum.msubmit.net/cgi-bin/main.plex. Go to Author Tasks and click the appropriate manuscript title to begin. The information you entered when you first submitted the paper will be displayed; update this as necessary. Note the following requirements:

• Upload point-by-point responses to the issues raised by the reviewers in a file named "Response to Reviewers," NOT in your cover letter.
• Upload a compare copy of the manuscript (without figures) as a "Marked-Up Manuscript" file.
• Upload a clean .DOC/.DOCX version of the revised manuscript and remove the previous version.
• Each figure must be uploaded as a separate, editable, high-resolution file (TIFF or EPS preferred), and any multipanel figures must be assembled into one file.
• Any supplemental material intended for posting by ASM should be uploaded with their legends separate from the main manuscript. You can combine all supplemental material into one file (preferred) or split it into a maximum of 10 files with all associated legends included.

For complete guidelines on revision requirements, see our Submission and Review Process webpage. Submission of a paper that does not conform to guidelines may delay acceptance of your manuscript.

Data availability: ASM policy requires that data be available to the public upon online posting of the article, so please verify all links to sequence records, if present, and make sure that each number retrieves the full record of the data. If a new accession number is not linked or a link is broken, provide Spectrum production staff with the correct URL for the record. If the accession numbers for new data are not publicly accessible before the expected online posting of the article, publication may be delayed; please contact production staff (Spectrum@asmusa.org) immediately with the expected release date.

Publication Fees: For information on publication fees and which article types are subject to charges, visit our website. If your manuscript is accepted for publication and any fees apply, you will be contacted separately about payment during the production process; please follow the instructions in that e-mail. Arrangements for payment must be made before your article is published.

ASM Membership: Corresponding authors may join or renew ASM membership to obtain discounts on publication fees. Need to upgrade your membership level? Please contact Customer Service at Service@asmusa.org.

The ASM Journals program strives for constant improvement in our submission and publication process. Please tell us how we can improve your experience by taking this quick Author Survey.

Thank you for submitting your paper to Spectrum.

Sincerely,
Blaire Steven
Editor
Microbiology Spectrum

Response to Reviewers
Manuscript ID: Spectrum03506-25
Title: Study on the Construction of Algal-Bacterial Consortium Comprising *Pantoea vagans* ZHS-1 and *Nostoc* sp. GD2 and Its Growth-Promoting Effect on Rice

Dear Editor and Reviewers,
We would like to express our sincere gratitude to the editor and the reviewers for their valuable time and insightful comments on our manuscript. These comments have been extremely helpful in improving the quality and clarity of our work. We have carefully considered all the suggestions and have revised the manuscript accordingly. Below is our point-by-point response to each comment, along with a description of the changes made.

Reviewer #1
General Comment: The manuscript "Study on the Construction of Algo-fungus Consortium and Its Growth-promoting Effect on Rice" aimed to assess a bacteria + algae consortium regarding several biochemical characteristics in BG11 culture medium, in addition to gene expression/validation assays and a plant growth-promotion evaluation. Despite the idea and the potential of the data being good, several flaws in the manuscript must be addressed to deliver a more diligent report and broaden the paper's audience. My main concerns are methodological flaws or conceptual mistakes described in the next paragraphs.
Response: We sincerely thank the reviewer for the positive assessment of our study's potential and for the detailed and constructive comments. We have carefully addressed all the concerns raised and have revised the manuscript thoroughly to enhance its scientific rigor and clarity.

Abstract and Importance
Comment: The authors should include the scientific names of the bacteria and cyanobacterial strains.
Response: We agree with the reviewer. The scientific names of both strains have now been included in the Abstract, Importance and Keywords sections.
Changes in Manuscript:

- **Abstract (Lines 13-14):** "...comprising the potassium-solubilizing bacterium *Pantoea vagans* ZHS-1 and the nitrogen-fixing cyanobacterium *Desmonostoc muscorum* GD2."
- **Importance (Line 28-29):** "...composed of *Pantoea vagans* ZHS-1 and *Desmonostoc muscorum* GD2..."
- **Keywords (Line 36):** "**Keywords:** algal--bacterial consortium; *Pantoea vagans*; *Desmonostoc muscorum*; ..."

Introduction
Comment: The authors describe the overuse of chemicals in Chinese agriculture very well. However, the topic lacks information from other studies on algae and algae/bacteria inoculation in plants, showing positive results for plant growth and yield. After presenting these results, the authors would present previous results with ZHS-1 and GD2, pointing out that those previous studies with single inoculation (alongside the literature data) inspired the team to test them under coinoculation.
Response: We thank the reviewer for this valuable suggestion. We have expanded the Introduction

to include recent studies on algae-based fertilizers and algal-bacterial co-inoculation. We then present our previous findings with ZHS-1 and GD2, leading to the rationale for testing them in co-culture.

Changes in Manuscript (Introduction):

- Added references to Nguyen et al. (2025) and Yu et al. (2025) on algal fertilizers.
- Added a paragraph summarizing the previous characterization of ZHS-1 and GD2 (Lines 77-86).
- Revised the rationale to explicitly state that the limited effects of single strains in paddy soil inspired us to test them in a consortium (Lines 87-92).

Materials and Methods

2.1 Experimental strains and culture media

Comment: The authors must include the scientific names of both strains used in this manuscript. Instead of citing only their reference, the authors must include additional information on the previous characterization of both strains.

Response: We have revised this section to include the full scientific names and a brief summary of the key functional characteristics of each strain, as previously reported.

Changes in Manuscript (Lines 105-109):

"Strain ZHS-1 is elliptical rod-shaped and identified as *Pantoea vagans*, which possesses multiple functions of potassium solubilization, phosphate solubilization, and indole-3-acetic acid (IAA) secretion. Strain GD2 is a filamentous cyanobacterium identified as *Desmonostoc muscorum*, with a maximum nitrogenase activity of 75.33 U·mg^{-1} under optimal conditions, exhibiting a significant nitrogen-fixing function."

Scanning Electron Micrograph of ZHS-1 and GD2:

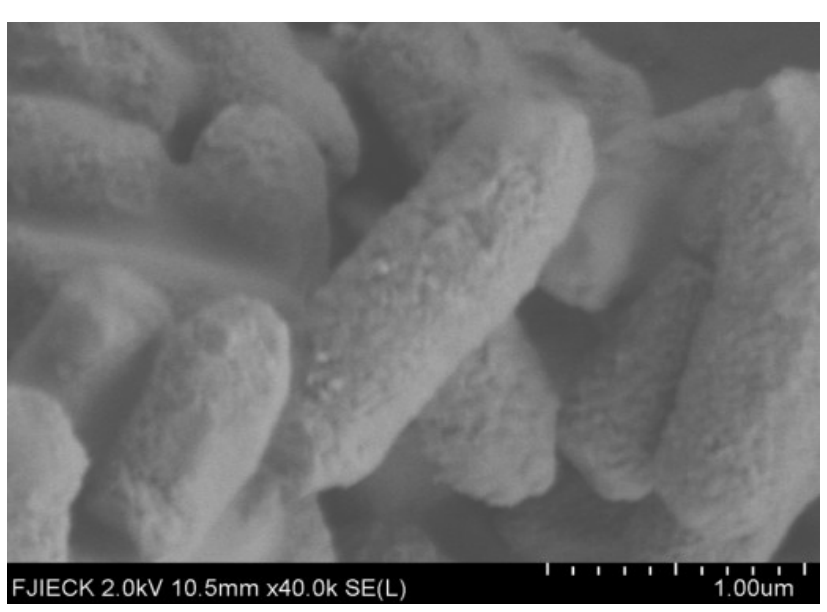

ZHS-1

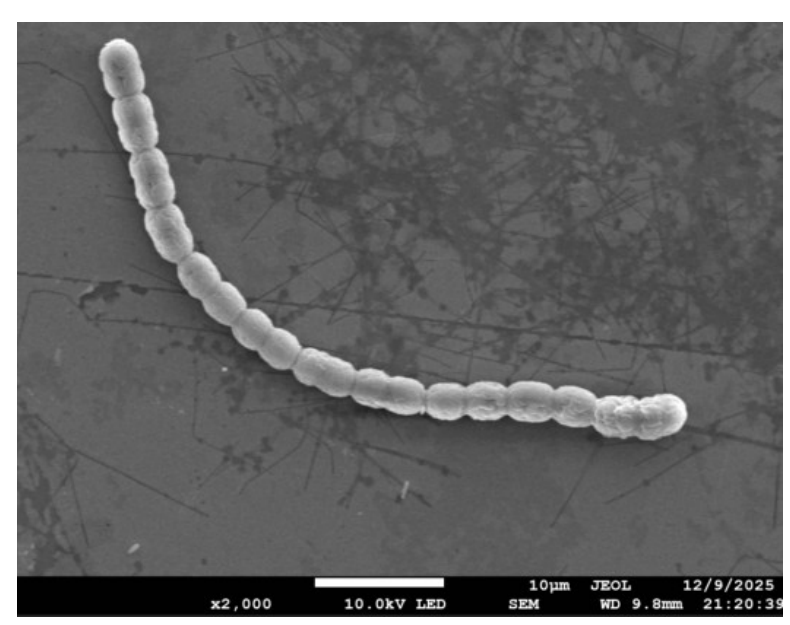

GD2

2.2.1 Algae-Bacteria co-cultivation experiment

Comment: It was not clear why the authors tested the different proportions ZHS-1/GD2 in the culture medium. As I understood, proportions ranged from 1:1 to 1:5 (ZHS-1:GD2). Why didn't they test the opposite proportions too (from 1:1 to 5:1, ZHS-1:GD2)? This information must be in the test explained in the motivation for testing this range of proportions.

Response: We thank the reviewer for this important question. We have now provided a clear scientific rationale for testing ratios with a higher proportion of GD2 relative to ZHS-1.

In algal-bacterial symbiotic systems, algal metabolites—particularly photosynthetically derived organic carbon—serve as the primary carbon source for heterotrophic bacteria. An excessively high proportion of bacteria could lead to intensified competition for limited nutrients, including nitrogen,

phosphorus, and algal-derived organic carbon. Such competition may result in nutrient deficiency and growth inhibition of the algae, potentially destabilizing the consortium. Therefore, to maintain a balanced and stable co-culture system, we focused on ratios where GD2 proportion is equal to or higher than that of ZHS-1.

Based on this rationale, five mass ratios (ZHS-1:GD2 = 1:1, 1:2, 1:3, 1:4, 1:5) were selected for systematic evaluation.

Changes in Manuscript (Lines 132-139):

"To evaluate the synergistic effects of ZHS-1 and GD2, five mass ratios (ZHS-1:GD2 = 1:1, 1:2, 1:3, 1:4, 1:5) were tested. The ratios were designed with a higher proportion of GD2 relative to ZHS-1, as algal metabolites typically serve as the primary carbon source for heterotrophic bacteria in algal-bacterial systems. An excessively high proportion of bacteria could lead to intensified competition for nutrients—such as nitrogen, phosphorus, and algal-derived organic carbon—potentially causing nutrient deficiency and growth inhibition of algae, and even disrupting the stability of the consortium (45, 46). The mixed cultures were inoculated into 100 mL BG11 liquid medium and incubated under the standard algal culture conditions for 7 days, with manual shaking every 12 h."

2.2.2 Screening for the optimal co-culture ratio

Comment: In lines 114-117, the authors argue that the measurement of N, K, and P in the supernatant indicated the N-fixation and the K and P solubilization potential of the consortia. However, they stated in the previous topic that after preparing the microbial mixes, they were "...inoculated into 100 mL of BG11 liquid medium to prepare the algae-bacteria seed suspension". Although the BG11 medium composition contains only 40 mg of K2HPO4 and no insoluble source of P and K, in this case, the micro-organisms have no insoluble P and K to dissolve. For N fixation, the ammonium in the medium is probably from the cell death and release, and not from the N fixation, since the fixed N will probably be used in full by the cyanobacterial filaments. The authors should reevaluate their argument for these measurements.

Response: We thank the reviewer for pointing out this conceptual issue. We have revised the description of these measurements to accurately reflect what they represent: the net release of nutrients into the medium, which results from combined processes including nutrient mobilization, metabolite release, and cellular turnover.

Regarding the nitrogen content determination, we agree with the reviewer that BG11 medium contains $NaNO_3$ as a nitrogen source, and that nitrogen accumulation in the supernatant may partially originate from cell death and release. Therefore, in the revised manuscript, we have clearly stated (Lines 156-158) that the mixtures were inoculated into BG11 liquid medium, and we noted that the observed nitrogen accumulation cannot be directly attributed to biological nitrogen fixation; it likely results from combined processes including metabolite release and cellular turnover.

Regarding the determination of phosphate-solubilizing capacity, we clearly indicated in the original manuscript (Line 170) that a **liquid inorganic phosphorus medium** was used, and its formulation was provided in **Section 2.2.1 Experimental Strains and Culture Media** (Lines 112–114). This medium contains insoluble tricalcium phosphate ($Ca_3(PO_4)_2$) as a phosphorus source, making it suitable for evaluating the phosphate-solubilizing ability of microorganisms.

Regarding the determination of potassium-solubilizing capacity, we clearly indicated in the original manuscript (Line 184) that a **modified potassium-solubilizing medium** was used, and its

formulation was provided in **Section 2.2.1 Experimental Strains and Culture Media** (Lines 116–120). This medium contains insoluble potassium feldspar powder as a potassium source, making it suitable for evaluating the potassium-solubilizing ability of microorganisms.

Therefore, the determinations of phosphate- and potassium-solubilizing capacities were conducted in specific media containing insoluble P/K sources, not in BG11 medium. We have clearly distinguished the different media used for each determination in the revised manuscript to avoid any confusion.

Changes in Manuscript:

- **Lines 152-156:** " These measurements reflect the net release of nutrients into the medium under different culture conditions. For phosphorus and potassium analyses, the media contained insoluble P/K sources, allowing assessment of solubilization capacity. For nitrogen analysis in BG11 medium, the measured accumulation results from combined processes including metabolite release and cellular turnover."
- **Lines 157-158:** "For nitrogen content analysis, the mixtures were inoculated into BG11 liquid medium..."
- **Line 171:** Clearly states that the phosphate-solubilizing capacity determination used liquid inorganic phosphorus medium.
- **Line 184:** Clearly states that the potassium-solubilizing capacity determination used modified potassium-solubilizing medium.
- **Results Section 3.1.2, Lines 411-413:** "Because BG11 medium contains a high concentration of $NaNO_3$, the observed nitrogen accumulation cannot be directly attributed to biological nitrogen fixation; it likely results from combined processes including metabolite release and cellular turnover."

2.3.1 Extracellular Polysaccharide Quantification

Comment: I was a little bit confused. So far, I know, the phenol-sulfuric acid method breaks down the EPS into its monosaccharides. For this reason, a standard curve of glucose can be used for quantification. The EPS is usually extracted with cold ethanol. Since the experiment has already been conducted, the authors should restate the aims and objectives of this analysis.

Response: We thank the reviewer for the opportunity to clarify. The aim of this analysis was to evaluate the potential of ZD-1 in biofilm formation and intercellular adhesion by quantifying the major components of EPS—extracellular polysaccharides and extracellular proteins. As EPS is primarily composed of these substances, their quantification provides a reliable assessment of EPS production, which is closely associated with biofilm formation and cell-cell interactions in algal-bacterial consortia.

Changes in Manuscript (Lines 191-192):

"To evaluate the potential of ZD-1 in biofilm formation and intercellular adhesion, extracellular polysaccharides—a major component of extracellular polymeric substances (EPS)—were quantified. "

2.3.2 Determination of Extracellular Protein Contents

Comment: Since the bacterial and algae growth had already been determined by plate count and chlorophyll content analysis, respectively, why is the quantification of proteins important at this point?

Response: We appreciate the reviewer's question. While plate counts and chlorophyll content provide information on biomass, they do not directly reflect metabolic activity or the production of functional extracellular compounds that mediate algal-bacterial interactions. Extracellular proteins, as key components of EPS, play essential roles in biofilm formation, cell adhesion, and intercellular communication. Therefore, quantifying extracellular protein content offers valuable insights into the physiological state of the consortium and the mechanisms underlying their synergistic interaction.

Accordingly, we have retained the determination of extracellular proteins in this study. In contrast, the analysis of intracellular protein content, which was considered redundant for the objectives of this work, has been completely removed from the manuscript to maintain focus and clarity.

Changes in Manuscript:

- A brief explanation of the rationale for extracellular protein quantification has been added at the beginning of section 2.3.2.
- All content related to intracellular protein determination has been deleted from the Materials and Methods, Results, and Figures sections.

2.3.4 Chemotaxis Assay

Comment 1: How could the authors determine the molarity of GD-2 secretions?

Response: The reviewer is correct; it is not possible to determine the molarity of a complex mixture like GD2 culture filtrate. We have corrected this terminology in the manuscript.

Changes in Manuscript (Lines 234-236):

"Sterile-filtered GD2 culture filtrate, as well as 1 M solutions of glucose, sucrose, maltose, trehalose, rhamnose, mannose, mannitol, and betaine prepared in BG11 liquid medium, were used as chemotactic solutions."

Comment 2: The method the authors use to assess bacterial mobility in the solutions is interesting at first glance. But, I think that the different amounts of bacteria observed in the different BG11 media supplied with the different C sources could be due to the bacterial preference for these sources and, consequently, their multiplication ability inside each C medium. Before using this method for the chemotaxis approach, it must be validated. I think a good way to do this is to inoculate the ZHS-1 bacteria into the same syringes (without the tip containing bacteria) and check whether the OD or plate counts change after an incubation period. Changes in initial bacterial counts indicate bacterial growth, which is particularly notable in GD-2 exudates.

Response: We thank the reviewer for this insightful comment, which touches upon a fundamental question in chemotaxis research: distinguishing between active migration and in situ proliferation.

We acknowledge that without direct growth controls, the observed differences in CFU could theoretically arise from differential bacterial multiplication within the test solutions. However, several lines of evidence support our interpretation that the observed RCI values primarily reflect chemotactic migration rather than differential growth:

First, if the RCI values were predominantly determined by bacterial growth rates in the test solutions, we would expect a positive correlation between the metabolic availability of the carbon source and the resulting CFU counts. Simpler sugars that are more readily metabolized should support faster bacterial growth and thus yield higher RCI values. However, our data show the opposite pattern: trehalose—a disaccharide that is typically metabolized more slowly than monosaccharides such as glucose—exhibited the highest RCI value (5.2), significantly exceeding

that of glucose (3.6). This inverse relationship between expected metabolic rate and observed bacterial accumulation strongly suggests that the differential CFU counts are driven by chemotactic attraction rather than differential growth.

Second, the RCI value for GD2 culture filtrate (4.7) fell between those of trehalose and rhamnose, which is consistent with a complex mixture containing multiple chemoattractants. If bacterial growth were the dominant factor, we would expect the complex filtrate to support intermediate growth rates—which it does—but this alone cannot explain the specific ranking of attractants that aligns with known chemotactic preferences of related bacteria reported in the literature [1-3].

Third, the capillary assay format itself limits the potential for substantial in situ growth during the 120-minute incubation period. Previous studies have demonstrated that under these conditions, the contribution of bacterial proliferation to the observed CFU differences is negligible compared to chemotactic accumulation [4,5].

We agree with the reviewer that direct growth controls would provide even stronger evidence. While such experiments were not included in the current study, we have now acknowledged this limitation in the revised Discussion section and propose that future investigations could include growth curves of ZHS-1 in each test solution to definitively separate chemotaxis from growth effects. Nevertheless, based on the reasoning above and the consistency of our results with established chemotaxis literature [1-5], we are confident that the observed RCI values reliably reflect chemotactic responses.

References Cited in This Response:

1. Adler J. A method for measuring chemotaxis and use of the method to determine optimum conditions for chemotaxis by Escherichia coli. Journal of General Microbiology. 1973;74(1):77-91.

2. Lewus P, Ford RM. Quantification of random motility and chemotaxis bacterial transport coefficients using individual-cell and population-scale assays. Biotechnology and Bioengineering. 2001; 75(3):292-304.

3. Ford RM, Lauffenburger DA. A simple expression for quantifying bacterial chemotaxis using capillary assay data: application to the analysis of enhanced chemotactic responses from growth-limited cultures. Mathematical Biosciences. 1992; 109(2):127-149.

4. Park H, Guet CC, Emonet T, Cluzel P. Fine-tuning of chemotactic response in E. coli determined by high-throughput capillary assay. Current Biology. 2011; 21(9):764-769.

5. Li J, Go AC, Ward MJ, Ottemann KM. The chemical-in-plug bacterial chemotaxis assay is prone to false positive responses. BMC Research Notes. 2010; 3:77.

2.5.1 Experimental Design and Treatments

Comment: In the plant assay, I can see a complete experiment with three treatments: 1) NPK, 2) NPK + ZD-1, and 3) ZD-1. The comparisons among these treatments are fair, since they have the same idea and basis (consortia and/or fertilization). The other two treatments N+ZHS-1 and PK+GD2 were probably added to assess the viability of bacteria and algae as sources of PK and N, respectively. However, they do not have the proper controls, which should be the treatments PK, N, GD2, and ZHS-1. In this case, comparing these two spare treatments with the first three is not fair, and the results will not be sufficiently confident to reveal the real impact of the microbes on supplementing fertilization in rice. Despite these two treatments, the authors should have included the 50% NPK and 50% NPK + ZD-1 treatments to assess the ability of the microbial consortia to

promote plant growth under limited fertilization conditions (if 50% NPK + ZD-1 = NPK, the result would be very good). As the experiment has already been done, I recommend analyzing, showing, and discussing only the three treatments cited above.

Response: We fully agree with the reviewer's assessment. We have followed the recommendation to focus our main analysis and conclusions on Treatments 1, 2, and 3. Treatments 4 and 5 are now explicitly described as exploratory groups in both the Methods and Results sections, and we have clearly stated that they cannot be directly compared with the other treatments due to the lack of proper controls. Their data are presented for transparency but are not used for statistical comparison or to draw main conclusions.

Changes in Manuscript:

- **Methods Section 2.5.1, Lines 270-282:** Added a paragraph explicitly stating the exploratory nature of T4 and T5 and the limitation in comparing them with T1-T3.
- **Results Section 3.4.1, Lines 542-545:** Added a similar caveat before presenting the T4/T5 data.
- **Results Sections 3.4.2 and 3.5:** The primary statistical comparisons and conclusions are now drawn from T1, T2, and T3.
- **Discussion Section, Lines 677-682:** Added a paragraph acknowledging this experimental design limitation.

2.7.2 and 2.7.3

Comment: No methods were cited at all.

Response: We have added the appropriate citations for the plant and soil nutrient determination methods.

Changes in Manuscript:

- **Line 344:** Added citation (27) for the nitrogen distillation method.
- **Line 457:** Added citation (28) for the phosphorus colorimetric method.
- **Line 461:** Added citation (29) for the soil available nitrogen method.
- **Line 494:** Added citation (30) for the soil total nitrogen method.

Results and Analysis

Comment 1: Is the title of this section right?

Response: We thank the reviewer for this important question. The reviewer is correct to question the title, and we have carefully reconsidered both the main section title and all subsection titles within the Results to ensure they accurately reflect the data presented.

First, regarding the main section title: We have changed it from "Results and Analysis" to the standard "RESULTS" as per journal guidelines (Line 385). This modification aligns with the formatting requirements of *Microbiology Spectrum* and is more consistent with standard scientific reporting conventions.

Second, and more importantly, we have thoroughly reviewed all subsection titles to ensure they accurately reflect the data presented and avoid any over-interpretation. In particular:

- Section 3.1.2: The original title "Nitrogen Fixation, Phosphorus Solubilization, and Potassium Solubilization by the Bacteria-Cyanobacteria Consortium" has been revised to "Nutrient Turnover Capacity of the Consortium" (Line 403). This change addresses the reviewer's valid concern (raised in Subtopic 2.2.2) that measurements in BG11 medium

cannot be directly attributed to biological nitrogen fixation, and now more accurately describes the net nutrient release observed in the supernatants.

- Section 3.2.2: The title has been revised to "Extracellular Polysaccharide, Extracellular Protein Production and Biofilm Formation" (Line 454) to clearly indicate which parameters were measured. The original title was imprecise and has been corrected to reflect that we quantified extracellular polysaccharides and extracellular proteins—major components of EPS—rather than total EPS.
- All other subsection titles have been checked for accuracy and consistency with the data presented, and any ambiguous or overstated terms have been revised accordingly.

These revisions ensure that the Results section, including all titles, accurately and transparently communicates our findings without overstatement.

Comment 2: Lack of statistical analysis for most of the biochemical or cell growth data in the manuscript. Proper statistics must be added and discussed.

Response: We thank the reviewer for pointing out this important omission. We have now performed statistical analyses for the quantitative data presented in the manuscript and have made corresponding revisions to the relevant figures and text.

Specifically:

- **Statistical analyses:** One-way analysis of variance (ANOVA) followed by Duncan's multiple range test was applied to all quantitative data, including growth curves (ZHS-1 CFU counts and GD2 chlorophyll content in **Fig. 1**), nutrient turnover measurements (nitrogen, phosphorus, and potassium concentrations in **Fig. 2**), EPS-related parameters (extracellular polysaccharide and extracellular protein contents in **Fig. 4A, B**), biofilm formation (OD_{590} values in **Fig. 4C**), plant nitrogen, phosphorus, and potassium accumulation (**Fig. 9**), and soil available nutrient contents (**Fig. 10**).
- **Significance indicators:** Different letters indicating statistically significant differences ($P < 0.05$) have been added to **Figures 1, 2, 4, 9, and 10**. These letters allow readers to readily identify significant differences between treatments or time points.
- **Figure legends:** The legends for all figures have been updated to explicitly state that data are presented as mean ± standard deviation (SD) and that different letters indicate significant differences at $P < 0.05$.
- **Results text:** The Results section has been revised to include appropriate references to the statistical findings. For example:
- In Section 3.1.1, we now state: "**on day 3, the colony count of ZHS-1 reached 26.67 × 10^5 CFU/mL, which was significantly higher than that of other ratios ($P < 0.05$; Fig. 1A).** "
- In Section 3.1.2, we now state: "**the 1:1 consortium displayed significantly higher soluble P than the ZHS-1 control from day 3 onward ($P < 0.05$; Fig. 2B).** "
- In Section 3.2.2, we now state: "**The biofilm-forming capacity of ZD-1 was significantly superior to that of single strains, with OD_{590} values 4.11-fold and 15.14-fold higher than those of ZHS-1 and GD2, respectively ($P < 0.05$; Fig. 4C).** "
- In Section 3.4.2, we now state: "**Among Treatments 1, 2, and 3, the NPK + ZD-1 treatment (T2) exhibited the highest overall nutrient accumulation, with significant differences compared to T1 and T3 ($P < 0.05$; Fig. 9).** "
- In Section 3.5, we now state: "**Notably, among Treatments 1, 2, and 3, the ZD-1**

treatment (T3) significantly increased soil available nitrogen, phosphorus, and potassium compared to T1 (NPK alone) ($P < 0.05$; Fig. 10). "

These additions ensure that the key conclusions drawn from the biochemical and plant growth data are statistically robust and properly discussed, addressing the reviewer's concern about the lack of statistical support in the original manuscript.

Suggestion: Traditional plant growth-promoting characteristics could be assessed in the single microbes and in the consortium. Characteristics such as auxin production and nitrogenase activity (acetylene reduction assay) could add valuable data to this manuscript.

Response: We thank the reviewer for this valuable suggestion. We agree that assessing traditional plant growth-promoting traits such as auxin production and nitrogenase activity would provide additional insights into the mechanisms underlying the beneficial effects of ZD-1.

However, the current study was designed to focus on the synergistic interactions between ZHS-1 and GD2 at the physiological, biochemical, and transcriptomic levels, with an emphasis on nutrient mobilization (nitrogen, phosphorus, and potassium) and plant growth promotion. While auxin production and nitrogenase activity are indeed important characteristics, they were beyond the scope of the present investigation.

Notably, the nitrogen-fixing capability of GD2 has been previously characterized in our laboratory, with a maximum nitrogenase activity of 75.33 $U \cdot mg^{-1}$ under optimal conditions, as reported in our earlier work (Wu et al., 2025). For ZHS-1, our previous studies have demonstrated its ability to secrete indole-3-acetic acid (IAA) (Tian et al., 2024).

We appreciate this suggestion and will consider incorporating these traditional plant growth-promoting assays in future studies to further elucidate the mechanisms of action of the ZD-1 consortium. We have now added a sentence in the Discussion section acknowledging this as a direction for future research (Lines 699-700).

Changes in Manuscript:

- **Discussion Section, Lines 699-700:** Added a sentence acknowledging that direct measurements of auxin production and nitrogenase activity in the ZD-1 consortium should be included in future studies to further elucidate the mechanistic basis of its growth-promoting effects.

Figure 1

Comment: The plot is confused. The best way to plot and understand it is to have the bacteria and algae in the same plot. So they may be separated by the proportions. The figure title must be self-explanatory.

Response: We thank the reviewer for this valuable suggestion regarding the presentation of Figure 1.

We understand the reviewer's recommendation to merge the two separate panels into a single integrated plot showing both bacterial and algal data together. However, after careful consideration, we have chosen to retain the current format with two separate panels (A and B) within the same figure, as we believe this presentation allows readers to clearly visualize the growth dynamics of each strain individually while still enabling easy comparison between different co-culture ratios.

To address the reviewer's concern about clarity, we have revised the figure title and legends to be more self-explanatory. The title now clearly states: "The growth of ZHS-1 and GD2 under different co-culture ratios." The legends have been updated to accurately describe each panel: "(A)

Colony-forming units (CFU) of ZHS-1; (B) Chlorophyll a content of GD2."

These revisions ensure that readers can clearly understand what each panel represents without ambiguity. We believe this addresses the reviewer's concern regarding the clarity of the figure title and legends, even while retaining the original panel format.

Changes in Manuscript (Figure 1, Lines 400-402):

"**Fig.1 The growth of ZHS-1 and GD2 under different co-culture ratios.** (A) Colony-forming units (CFU) of ZHS-1. (B) Chlorophyll a content of GD2."

We would like to express our sincere gratitude to the reviewer again for your careful observation and meticulous scrutiny, which helped us improve the clarity of our data presentation.

Subtopic 3.1.2 and Figure 2

Comment: These results cannot be presented as N fixation and PK solubilization for the reasons explained above (Subtopic 2.2.2). After elaborating on the methodology and objectives of this analysis, the authors will need to restructure how they present these data. Figure 2: The figure is poor and not readable.

Response: We have restructured the presentation of these data. The section title has been changed to "Nutrient Turnover Capacity," and the text has been revised to describe the observations without over-interpreting them as direct measures of fixation/solubilization. We have also generated a new, high-resolution version of Figure 2 with improved readability.

Changes in Manuscript:

- **Section Title, Line 403: "3.1.2 Nutrient Turnover Capacity of the Consortium"**
- **Lines 404-406:** "To evaluate nutrient transformation-related parameters in co-cultures with different ratios, the contents of total nitrogen (in BG11 medium), soluble phosphorus (in IPM) and soluble potassium (in MKSM) were monitored over 7 days."
- **Figure 2 (Line 426):** Replaced with a high-resolution, clearly labeled figure.

Subtopic 3.2.1 (Chemotaxis)

Comment: As discussed above (Subtopic 2.5), I am not quite sure if this result is due to the bacterial migration from one matrix to another or if it is a result of bacterial growth in the new BG11 medium. Before a validation experiment for this new approach, this data is not trustworthy, and if the authors can not assess or validate it now, they should not use this data in this manuscript.

Response: We thank the reviewer for this comment. As detailed in our response to the methodological concerns raised in Subtopic 2.3.4, we have provided multiple lines of evidence supporting the reliability of our chemotaxis data, including the inverse relationship between expected metabolic rates and observed RCI values, as well as supporting literature demonstrating the validity of this approach [1-5]. Based on these considerations, we are confident that the data presented in Fig. 3 reliably reflect genuine chemotactic responses and should be retained in the manuscript.

Subtopic 3.2.2

Comment 1: The intracellular and extracellular protein increase is expected, in my point of view. The extracellular protein content likely comes from cell death and lysis, and if there are more live cells, there should be more dead ones as well.

Response: We thank the reviewer for this insightful comment. We agree that extracellular proteins

can originate from cell death and lysis. However, in this study, we focused specifically on extracellular protein content as an indicator of metabolic activity and the production of functional extracellular compounds that mediate algal-bacterial interactions, rather than as a measure of total protein synthesis.

It is important to clarify that intracellular protein content was not determined in this study. As described in our response to the methodological comments in Section 2.3.2, all content related to intracellular protein measurement has been removed from the manuscript to maintain focus and clarity. The extracellular protein data presented in Fig. 4B reflect the proteins actively secreted or released into the culture supernatant by ZHS-1, GD2, and the ZD-1 consortium.

While we acknowledge that cell lysis may contribute to extracellular protein levels, our transcriptomic data provide additional insights. The upregulation of *pheT* (phenylalanyl-tRNA synthetase) in GD2 and ribosomal genes in both strains under co-culture conditions (Section 3.3) suggests enhanced protein synthesis capacity, which may support increased production and active secretion of extracellular proteins. This interpretation is consistent with the observed synergistic enhancement of biofilm formation (Fig. 4C) and EPS production (Fig. 4A) in ZD-1.

We have revised the Results section to present the extracellular protein data without over-speculation, and we have incorporated this nuanced discussion of the potential sources and functional significance of extracellular proteins in the revised Discussion section.

Comment 2: Lines 416-417: These assumptions are speculative, not the manuscript data.

Response: We have removed the speculative statements from the Results section.

Changes in Manuscript (Lines 468-470):

The original speculative sentences have been removed. The text now simply reports the findings: "The extracellular polysaccharide contents... increased with growth... The biofilm-forming capacity of ZD-1 was significantly superior..."

Comment 3: Regarding the consortia's biofilm-forming capacity, a discussion of multispecies biofilms is worth noting, supported by literature data.

Response: We have expanded the discussion of biofilm formation in the Discussion section, incorporating literature on multispecies biofilms.

Changes in Manuscript (Discussion, Lines 648-650):

"...thereby facilitating the formation of biofilms from the extracellular substances of the two strains, which is the key to their non-antagonistic and mutually promoting growth (59)."

Subtopic 3.5.2

Comment: Could the nif genes upregulation in ZD-1 occur due to the higher algal growth in the medium? In this case, more cells and more gene expression compared to GD2 alone.

Response: This is a valid consideration. The reviewer is correct that increased biomass could contribute to higher overall gene expression. However, our RT-qPCR data were normalized to reference genes and represent *relative expression levels per cell*. Therefore, the observed upregulation of *nif* genes in ZD-1 compared to GD2 alone indicates a true increase in transcriptional activity per cell, not just a biomass effect. We have added a brief clarification in the text to address this point.

Changes in Manuscript (Lines 537-540):

"The upregulation of these genes, as determined by RT-qPCR with normalization to reference genes, indicates a true increase in transcriptional activity per cell in the co-culture context, rather than being

solely a consequence of increased biomass."

Figures 5, 6, and 7

Comment: The figures are not readable.

Response: We have generated new, high-resolution versions of Figures 5, 6, and 7. The resolution, font sizes, and labels have been optimized for clarity.

Subtopic 3.6, 3.7, and 3.8 (Plant Data)

Comment: As I pointed out in subtopic 2.7.1, the plant assay must be reanalyzed and the results presented again. After the new presentation, and in a more straightforward and assertive manner, this subtopic will be better aligned with the manuscript as a whole and will highlight the potential of bacterial consortia through fair analysis and comparisons.

Response: As detailed in our response to the comment on 2.7.1, we have restructured the presentation of the plant data. The primary analysis and conclusions are now focused on Treatments 1, 2, and 3. The data from Treatments 4 and 5 are presented as exploratory observations with appropriate caveats.

Discussion

Comment: The discussion is vague. The authors cite several references that talk to each other but not to the manuscript's data. In this discussion, the authors must use the references to compare them with their data and the data's correlation. In this section, the authors could correlate their data (among others): 1) optimization of ZD-1 consortium and the single strains behaviour in all different tested conditions; 2) growth (the authors call "chemotaxis") of ZHS-1 in GD2 exudates and biofilm formation; 3) consortium-growth and gene expression; 4) EPS production x biofilm formation x plant growth promotion. These results must "talk" to each other and use the references as support for your results. After the restructuring of the data presentation, the changes in the discussion will be easier to make.

Response: We thank the reviewer for this insightful comment, which has guided us to thoroughly restructure the Discussion section to ensure that all claims are firmly grounded in our data and supported by relevant literature.

We have now revised the Discussion section to establish clear connections between our key findings and the existing literature, following the four areas suggested by the reviewer:

1. Consortium optimization and single-strain behavior: In the first paragraph, we discuss how the optimal 1:1 ratio, identified through growth dynamics (Fig. 1) and nutrient turnover data (Fig. 2), reflects a balance between mutualism and competition. This finding is contextualized within the framework of algal-bacterial interactions, supported by literature on metabolic complementarity and nutrient exchange (e.g., Ramanan et al., 2016; Matthews et al., 2023).

2. Chemotaxis and biofilm formation: In the second paragraph, we directly link the strong chemotactic response of ZHS-1 to GD2 exudates (RCI = 4.7, Fig. 3) with the enhanced extracellular polysaccharide production and biofilm formation in ZD-1 (Fig. 4). This connection is supported by studies demonstrating that chemotaxis-mediated attraction facilitates initial colonization and subsequent biofilm development in algal-bacterial systems (e.g., Raina et al., 2023; Clerc et al., 2023).

3. Consortium growth and gene expression: In the third paragraph, we correlate the

transcriptomic data (Fig. 5-7) with the observed phenotypic outcomes. Specifically, we link the upregulation of *galF* in ZHS-1 to increased extracellular polysaccharide production (Fig. 4A), and the upregulation of *nifH*, *nifD*, and *nifV* in GD2 to enhanced nitrogen availability (Fig. 2A, 9A). The upregulation of ribosomal genes and *pheT* is discussed in the context of enhanced protein synthesis capacity, which aligns with the increased extracellular protein content observed in ZD-1 (Fig. 4B). The RT-qPCR data normalized to reference genes confirm that these changes reflect true increases in transcriptional activity per cell rather than merely increased biomass.

4. EPS production, biofilm formation, and plant growth promotion: Throughout the discussion, we synthesize these findings to propose a mechanistic model wherein chemotaxis-driven recruitment leads to robust biofilm formation, creating a protective and efficient microenvironment that facilitates nutrient exchange and metabolic synergy. This ultimately results in enhanced plant growth and soil nutrient activation (Fig. 8-10). This model is supported by literature on the role of biofilms in plant-microbe interactions (e.g., Zhang et al., 2020; Balducci et al., 2023).

Additionally, we have added a paragraph at the end of the Discussion acknowledging the limitations of this study, including the absence of direct measurements of traditional plant growth-promoting traits such as auxin production and nitrogenase activity, while noting that these have been previously characterized in our laboratory (Tian et al., 2024; Wu et al., 2025) and should be addressed in future studies. We also acknowledge the limitation regarding the potential contribution of cell lysis to extracellular protein levels, while presenting transcriptomic evidence supporting active secretion.

These revisions ensure that the Discussion section now provides a coherent narrative that integrates all major findings, demonstrates how our data "talk" to each other, and is firmly supported by relevant literature.

We hope that these revisions meet the approval of the editor and reviewers. We are grateful for the opportunity to improve our manuscript and believe it is now significantly stronger.

Sincerely,

The Authors

Re: Spectrum03506-25R2 (**Study on the Construction of Algal - Bacterial Consortium Comprising *Pantoea vagans* ZHS-1 and *Nostoc* sp. GD2 and Its Growth-Promoting Effect on Rice**)

Dear Dr. Lei Wu:

Thank you for the modifications. I am now pushing this manuscript forward to acceptance.

Your manuscript has been accepted, and I am forwarding it to the ASM production staff for publication. Your paper will first be checked to make sure all elements meet the technical requirements. ASM staff will contact you if anything needs to be revised before copyediting and production can begin. Otherwise, you will be notified when your proofs are ready to be viewed.

Data Availability: ASM policy requires that data be available to the public upon online posting of the article, so please verify all links to sequence records, if present, and make sure that each number retrieves the full record of the data. If a new accession number is not linked or a link is broken, provide production staff with the correct URL for the record. If the accession numbers for new data are not publicly accessible before the expected online posting of the article, publication may be delayed; please contact ASM production staff immediately with the expected release date.

Publication Fees: For information on publication fees and which article types have charges, please visit our website. We have partnered with Copyright Clearance Center (CCC) to collect author charges. If fees apply to your paper, you will receive a message from no-reply@copyright.com with further instructions. For questions related to paying charges through RightsLink, please contact CCC at ASM_Support@copyright.com or toll free at +1-877-622-5543. CCC makes every attempt to respond to all emails within 24 hours.

ASM Membership: Corresponding authors may join or renew ASM membership to obtain discounts on publication fees. Need to upgrade your membership level? Please contact Customer Service at Service@asmusa.org.

Embargo Policy: A press release may be issued as soon as the manuscript is posted on the Spectrum Latest Articles webpage. The corresponding author will receive an email with the subject line "ASM Journals Author Services Notification" when the article is available online.

The ASM Journals program strives for constant improvement in our submission and publication process. Please tell us how we can improve your experience by taking this quick Author Survey.

Thank you for submitting your paper to Spectrum.

Sincerely,
Blaire Steven
Editor
Microbiology Spectrum
