# Supplementary material for: Study on the construction of algal–bacterial consortium comprising Pantoea vagans ZHS-1 and Nostoc sp. GD2 and its growth-promoting effect on rice
Source: Microbiol Spectr. 2026 Aug 31;14(10):e03506-25. doi: 10.1128/spectrum.03506-25 (PMC13688834; doi:10.1128/spectrum.03506-25)
Supplement: Supplementary file 2 [file spectrum.03506-25-s0001.docx]

Supplementary Fig.1 Relative expression level of differentially expressed genes by RT-qPCR

Based on the aforementioned KEGG and GO enrichment results, the gene expression levels from the RNA sequencing data were validated by performing RT-qPCR analysis on 10 selected differentially expressed genes (DEGs). The 10 DEGs were as follows: *pasJ*, *apcB*, *psbU*, *apcD*, *rplJ*, *rpsB*, *rpsL*, *rpmB*, *rpsF*, and *rplK*. Quantitative primers were designed based on the sequences of these 10 DEGs, and RT-qPCR analysis was subsequently conducted. The results were consistent with the transcriptome data: the expressions of genes *apcD*, *rpsL*, *rpmB*, *rpsF*, and *rplK* were significantly upregulated, while the expressions of genes *pasJ*, *apcB*, *psbU*, *rplJ*, and *rpsB* were significantly downregulated (Figure 10), which aligned with the transcriptome results.
