# Supplementary material for: Study on the construction of algal–bacterial consortium comprising Pantoea vagans ZHS-1 and Nostoc sp. GD2 and its growth-promoting effect on rice
Source: Microbiol Spectr. 2026 Aug 31;14(10):e03506-25. doi: 10.1128/spectrum.03506-25 (PMC13688834; doi:10.1128/spectrum.03506-25)
Supplement: Supplementary file 3 [file spectrum.03506-25-s0002.docx]

Supplementary Tab.1 Reverse transcription system

| Components | dosages |
| --- | --- |
| Total DNA | ≤1 μg/≤100 ng |
| 5xTransScript^®^Uni All-in-One SuperMix for qPCR | 4 μL |
| TransScript® Uni All-in-One Enzyme Mix | 1 μL |
| RNase-free Water | final volume adjusted to 20 μL |
