# Supplementary material for: Study on the construction of algal–bacterial consortium comprising Pantoea vagans ZHS-1 and Nostoc sp. GD2 and its growth-promoting effect on rice
Source: Microbiol Spectr. 2026 Aug 31;14(10):e03506-25. doi: 10.1128/spectrum.03506-25 (PMC13688834; doi:10.1128/spectrum.03506-25)
Supplement: Supplementary file 4 [file spectrum.03506-25-s0003.docx]

Supplementary Tab.2 qPCR primer sequences

| Primers | F/R（5’- 3’） |
| --- | --- |
| 16S rDNA | GGACGGGTGAGTAACGCGTA/CCCATTGCGGAAAATTCCCC |
| *pasJ* | ATGGCAGACAAAGGCGACAACTC/TGTGATTGTCAGCCAGATGGTAGC |
| *psbU* | CTTGGTTGCTGGGGATGGTTGG/TGTCTGCGCTATTCTGCCGTTC |
| *apcB* | TAATGCAGCCGGACTTGTCAAGC/CCGCAAACAAGCCGCATAACG |
| *apcD* | ACAACTCATTGGGCGTTCCTGTC/TGCAGCTTCAGCAGCGTCTTC |
| *rpsL* | CTGCCAGGTGTGCGTTACCAC/GCTTCTTCACGCCGTACTTGGAG |
| *rpmB* | CCTTCCGAACCTGCACTCACAC/CTCACCACGGGCACGCATATC |
| *rpsB* | CAGGTAACGACGATGCGATCCG/CTTCAGCTTGCTCAGCCAGGTC |
| *rpsF* | GGCACGATTCACCGTCTGGAAG/GGCGTCGTTGAAGCGGAAGTTC |
| *rplK* | CGGGCATCAAGTCTGGTTCTGG/GTCATGTCCGCAGCTTTGGTTTC |
| *rplJ* | CAACGCTGCCGACCTACGAAG/GCCAGAGTGCGGACCAGTTTG |
