# Supplementary material for: Study on the construction of algal–bacterial consortium comprising Pantoea vagans ZHS-1 and Nostoc sp. GD2 and its growth-promoting effect on rice
Source: Microbiol Spectr. 2026 Aug 31;14(10):e03506-25. doi: 10.1128/spectrum.03506-25 (PMC13688834; doi:10.1128/spectrum.03506-25)
Supplement: Supplementary file 5 [file spectrum.03506-25-s0004.docx]

Supplementary Tab.3 qPCR reaction system

| Components | Volume | Final concentration |
| --- | --- | --- |
| Template | 2 μL | - |
| Forward primer | 0.4 μL | 0.2 μM |
| Reverse primer | 0.4 μL | 0.2 μM |
| 2xPerfectStartGreen®qPCR SuperMix | 10 μL | 1 x |
| Passive Reference Dye(50x)(optional) | 0.4 μL | 1 x |
| Nuclease-free Water | 6.8 μL | - |
| Total volume | 20 μL | - |
