# Supplementary material for: Study on the construction of algal–bacterial consortium comprising Pantoea vagans ZHS-1 and Nostoc sp. GD2 and its growth-promoting effect on rice
Source: Microbiol Spectr. 2026 Aug 31;14(10):e03506-25. doi: 10.1128/spectrum.03506-25 (PMC13688834; doi:10.1128/spectrum.03506-25)
Supplement: Supplementary file 6 [file spectrum.03506-25-s0005.docx]

Supplementary Tab.4 qPCR amplification condition

| Temperature | Time | Cycle |
| --- | --- | --- |
| 94 ℃ | 30 s | 1 |
| 94 ℃ | 5 s |  |
| 55 ℃ | 15 s | 45 cycles |
| 72 ℃ | 10 s |  |
